# Supplementary material for: Computationally unmasking each fatty acyl C=C position in complex lipids by routine LC-MS/MS lipidomics
Source: Nat Commun. 2025 Aug 11;16:7277. doi: 10.1038/s41467-025-61911-x (PMC12340080; doi:10.1038/s41467-025-61911-x)
Supplement: Supplementary file 1 — Supplementary Information [file 41467_2025_61911_MOESM1_ESM.pdf]

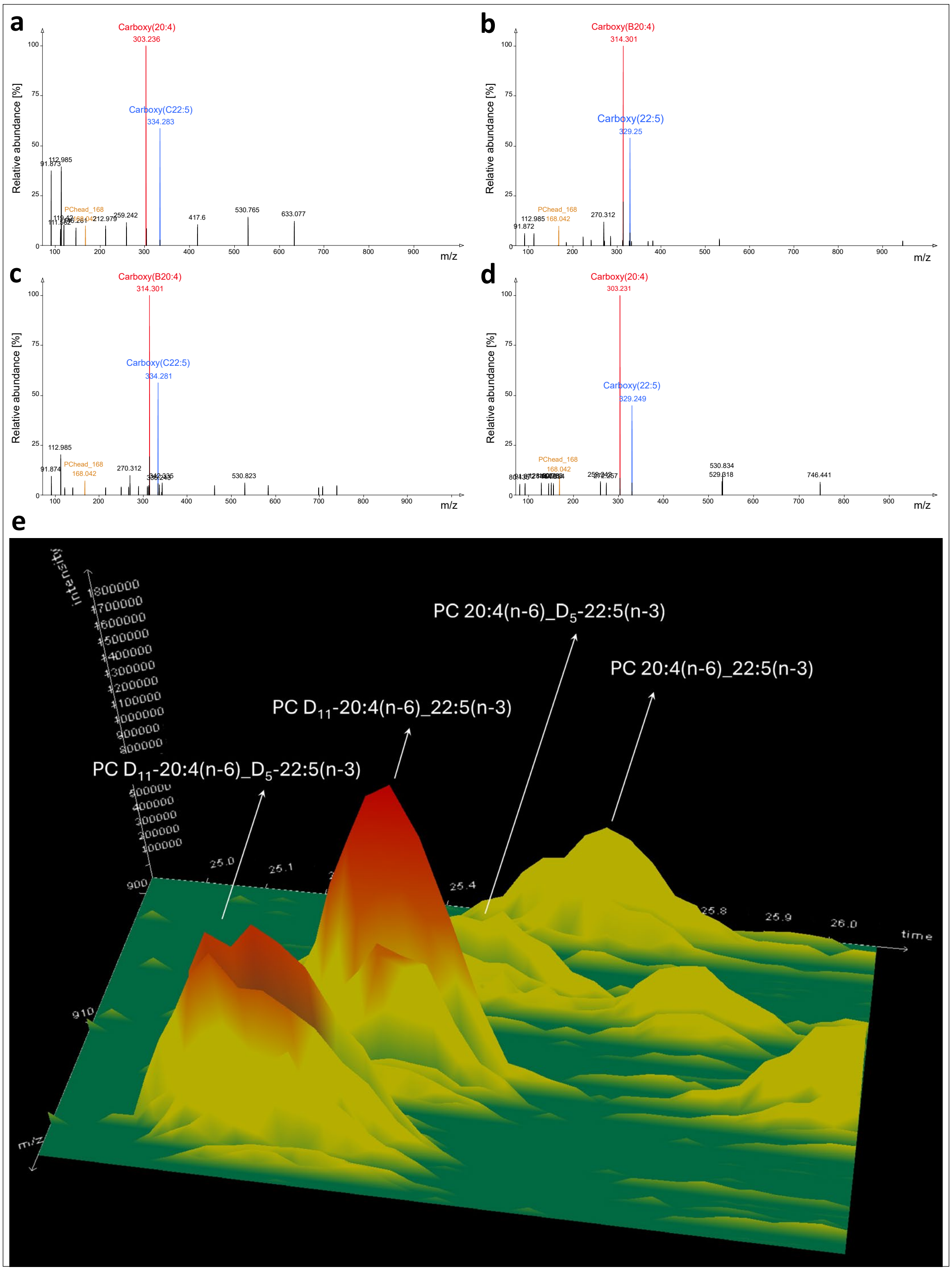

Supplementary Fig. 1: Example of all possible SIL versions of PC 20:4(n-6)_22:5(n-3) aggregated to a single RT-DB entry after correction of the isotope effect on RT.

Spectral evidence of all four isotopologues of PC 20:4(n-6)_22:5(n-3) detected as $[M+HCOO]^-$ in an extract of RAW264.7 cells supplemented with D_5-18-3(n-3) and D_{11}-18-2(n-6) (file: 03_d5-18-3(n-3)+d11-18-2(n-6)_60min) using a 60-minute chromatography. The RTs of all three SIL isotopologues are predicted at the measured RT of the unlabeled PC 20:4(n-6)_22:5(n-3) and automatedly consolidated in one RT-DB entry. **a** Identification of PC 20:4(n-6)_D_5-22:5(n-3) at RT = 25.47 min (precursor *m/z* = 905.607), RT offset to unlabeled species = 0.23 min. **b** Identification of PC D_{11}-20:4(n-6)_22:5(n-3) at RT = 25.44 min (precursor *m/z* = 911.645), RT offset to unlabeled species = 0.27 min. **c** Identification of double labeled PC D_{11}-20:4(n-6)_D_5-22:5(n-3) at RT = 25.24 min (precursor *m/z* = 916.676), RT offset to unlabeled species = 0.46 min. **d** Identification of unlabeled PC 20:4(n-6)_22:5(n-3) at RT = 25.70 min (precursor *m/z* = 900.576). **e** Peaks of the four isotopologues described above in LDA 3D GUI, visualizing the RT and *m/z* offset caused by SIL.

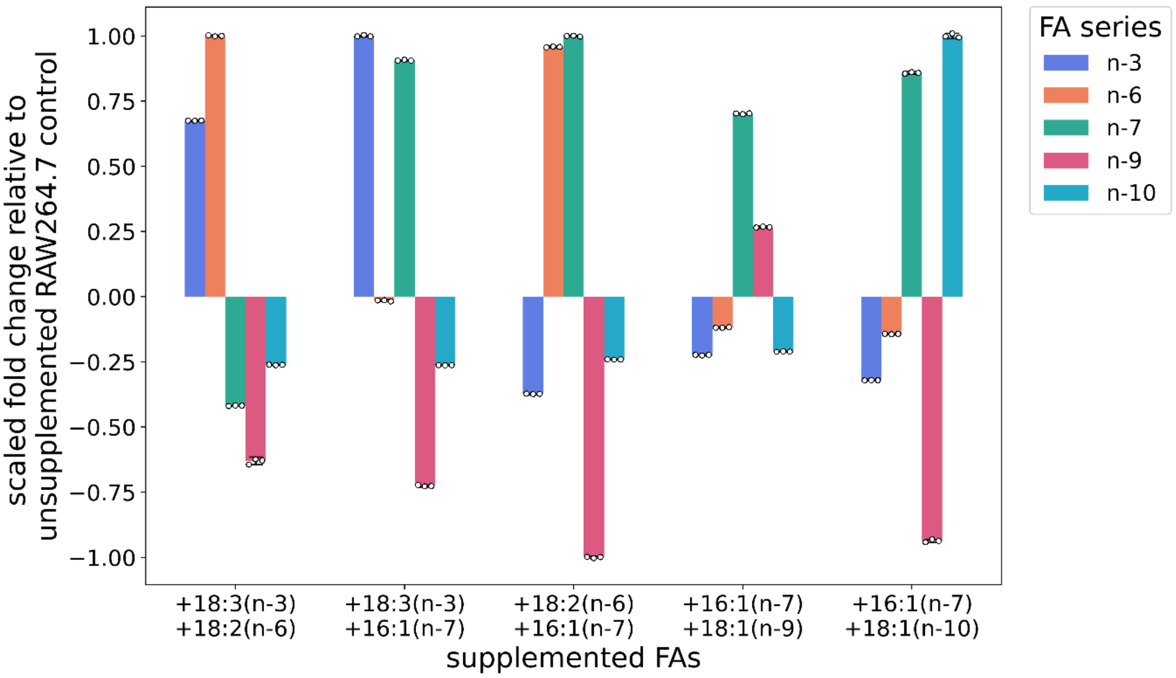

Supplementary Fig. 2: Relative increase of corresponding ω-position FA series based on supplementation of native FAs in pairs.

RAW264.7 cells were supplemented with five different pairs of unlabeled FAs 18:3(n-3), 18:2(n-6), 16:1(n-7), 18:1(n-9), and 16:1(n-10) (x-axis). Cell extracts were measured in technical triplicates (n=3) by RPLC-MS/MS, and intact phospholipids were fully automatedly analyzed with LC=CL (no manual curation). A total of 2074 ω-resolved lipids were identified in this analysis. Relative phospholipid FA content for each ω-position series (n-3, n-6, n-7, n-9 and n-10) was computed as a fold-change relative to the unsupplemented RAW264.7 cell extract. Y-axis was scaled to the highest absolute fold-change value for each FA series. Error bars represent the standard deviation. Each experiment results in an increase of the FA series corresponding to the ω-positions of the supplemented FA precursors as detected by LC=CL. I.e., when 18:3(n-3) and 18:2(n-6) are supplemented, the "n-3" and "n-6" FA series exhibit a selective increase. LC=CL demonstrated the ability to accurately distinguish complex lipid isomers that differ by just a singular ω-position (e.g., n-6 and n-7 or n-9 and n-10). Source data are provided as a Source Data file.

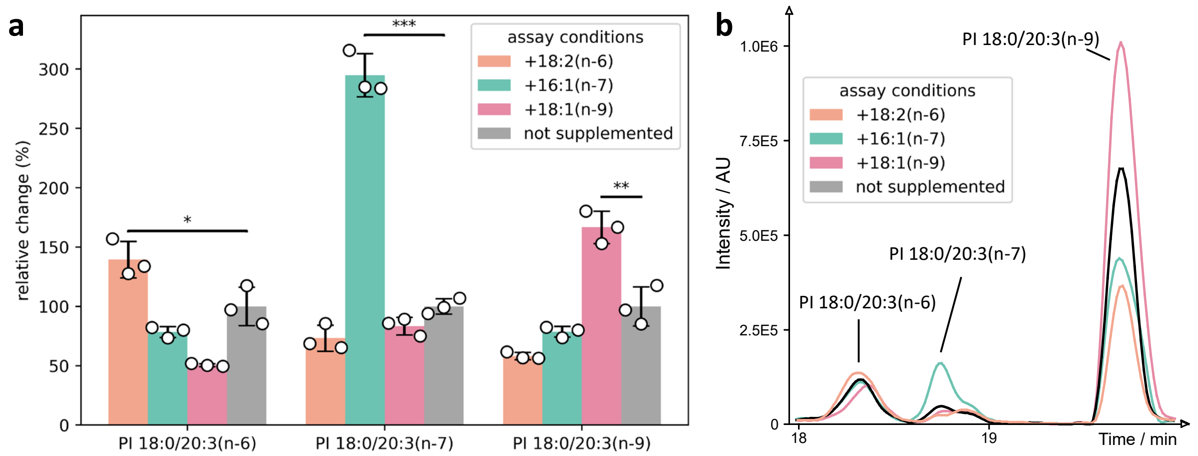

Supplementary Fig. 3: Relative abundance increase of PI species with corresponding ω-positions as identified by LC=CL following supplementation of single native FAs.

RAW264.7 cells were supplemented with the native FAs 18:2(n-6), 16:1(n-7), and 18:1(n-9). Cell extracts were measured in biological triplicates (n=3) by RPLC-MS/MS, and intact phospholipids were fully automatedly analyzed by LC=CL which identified the three observed PI 18:0/20:3 isomers as n-6, n-7, and n-9, indicated by orange, green and pink color, respectively. **a** Relative changes in abundance for each isomer was calculated in comparison to unsupplemented RAW264.7 cell extract (indicated in grey color). Error bars represent the standard deviation. Each experiment results in an increase of the FA series corresponding to the ω-positions of the supplemented FA precursors as detected by LC=CL. p-Values for the two-sided statistical t-test for the comparisons of the supplemented assays with the data of not supplemented RAW264.7 cell extract. The shorthand notations for the p-values for the two-sided statistical t-tests comparing the respective supplemented assays with the data of not supplemented RAW264.7 cell extract represent the following significance thresholds: *** < 0.001, ** < 0.01, * < 0.05. Source data are provided as a Source Data file. **b** Relative changes in abundance for each isomer illustrated qualitatively in an overlay of the chromatograms. PI 18:0/20:3(n-6) was detected at RT=18.28 min, PI 18:0/20:3(n-7) at RT=18.74 min, PI 18:0/20:3(n-9) at RT=19.67 min.

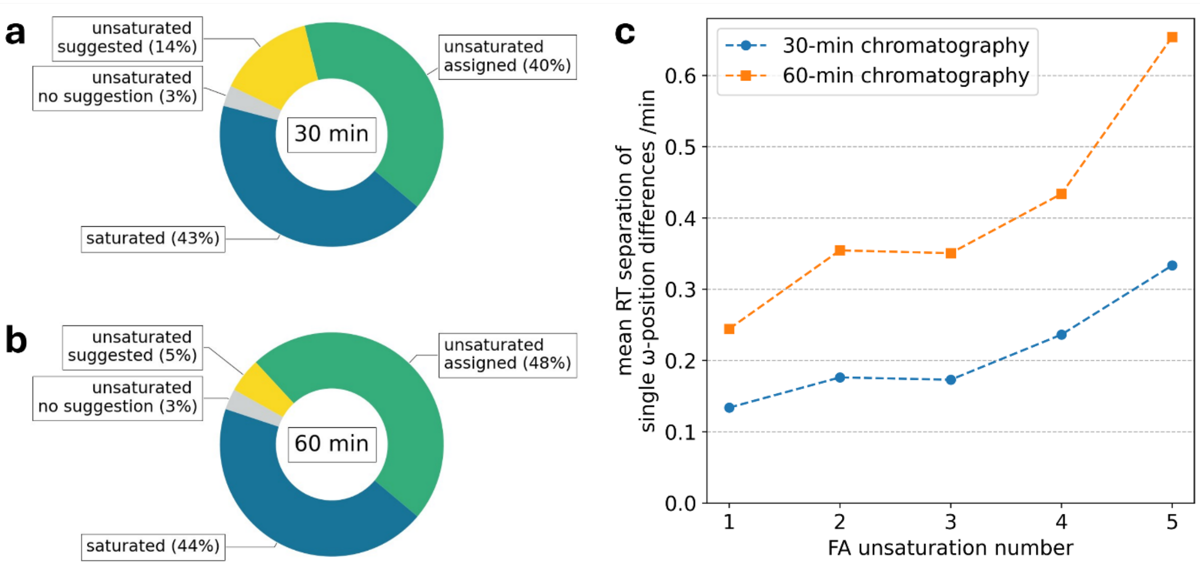

Supplementary Fig. 4: Number of automated ω-identifications by LC=CL correlates with the discriminative power of the chromatography.

Total FA content in the human plasma phospholipidome annotated by LC=CL in a 30-minute (**a**) and 60-minute (**b**) experiment, respectively. LC=CL in combination with LDA provided full annotation up to ω-level for approximately 97% of the total FA content (corresponds to 95% of unsaturated FAs). The 'unsaturated suggested' portion did not meet the stringent criteria for automated identifications. The increased number of suggested species in the 30-minute experiment reflects the lower discriminative power of the shorter chromatography. **c** The discriminative power of the employed chromatographic methods increases as a function of FA unsaturation. The mean RT separation of ω-position isomers listed in the RT-DB was computed for single ω-position differences, i.e. the RT difference between isomers was divided by their ω-position difference (3 for n-3 species compared to n-6 species). Source data are provided as a Source Data file.

Supplementary Fig. 5: Spectral evidence for FAs not previously documented in RAW264.7 cells.
Spectral evidence for each of the 20 FAs that to the best of our knowledge were documented for the first time in RAW264.7 cells by our study. Where available, MS^n spectra proving the respective unlabeled isotopologues at the predicted RTs on the 60-minute chromatography are shown in addition to the SIL species. **aa-al**, spectral evidence for n-3 series FAs; **ba-bj**, spectral evidence for n-6 series FAs; **ca-cf**, spectral evidence for n-7 series FAs; **da-dj**, spectral evidence for n-9 series FAs; **ea**, spectral evidence for one n-10 series FA.

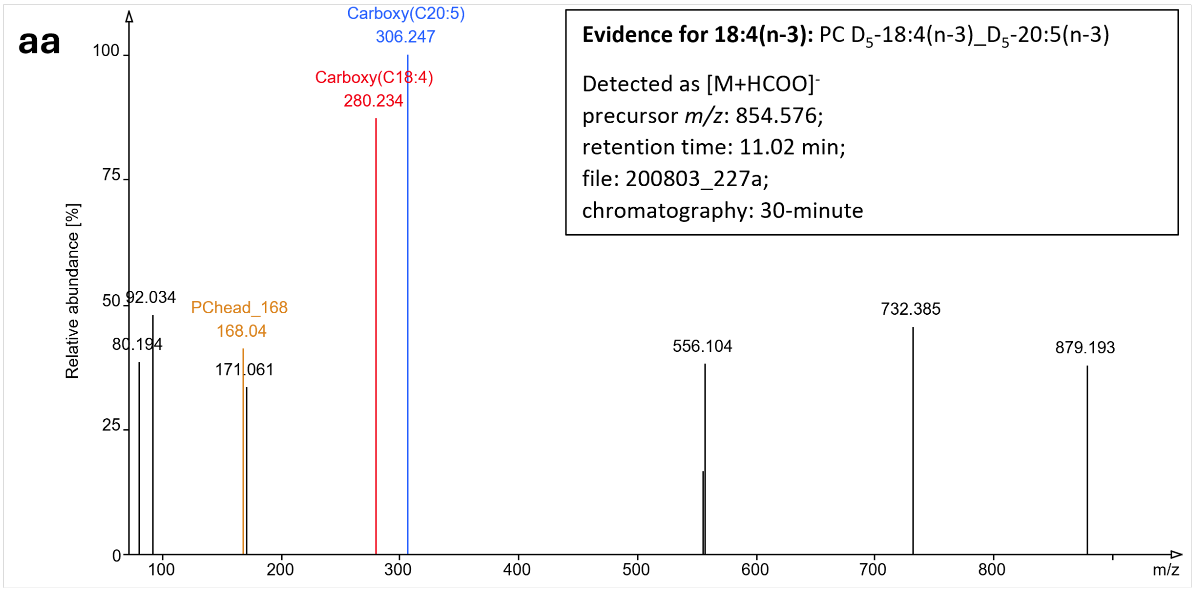

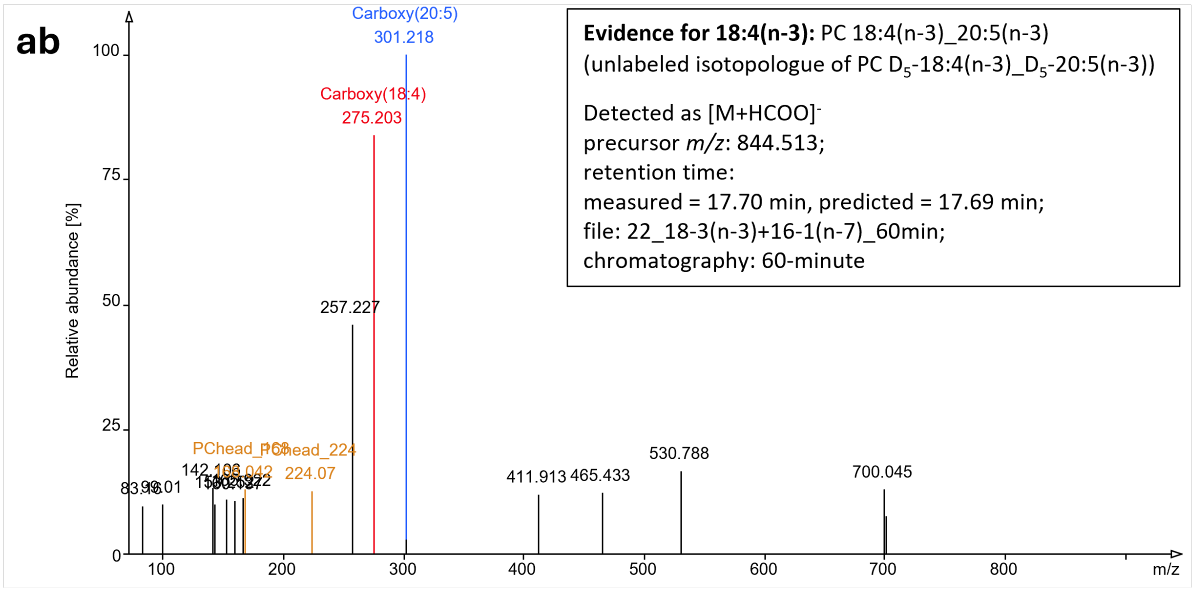

ac

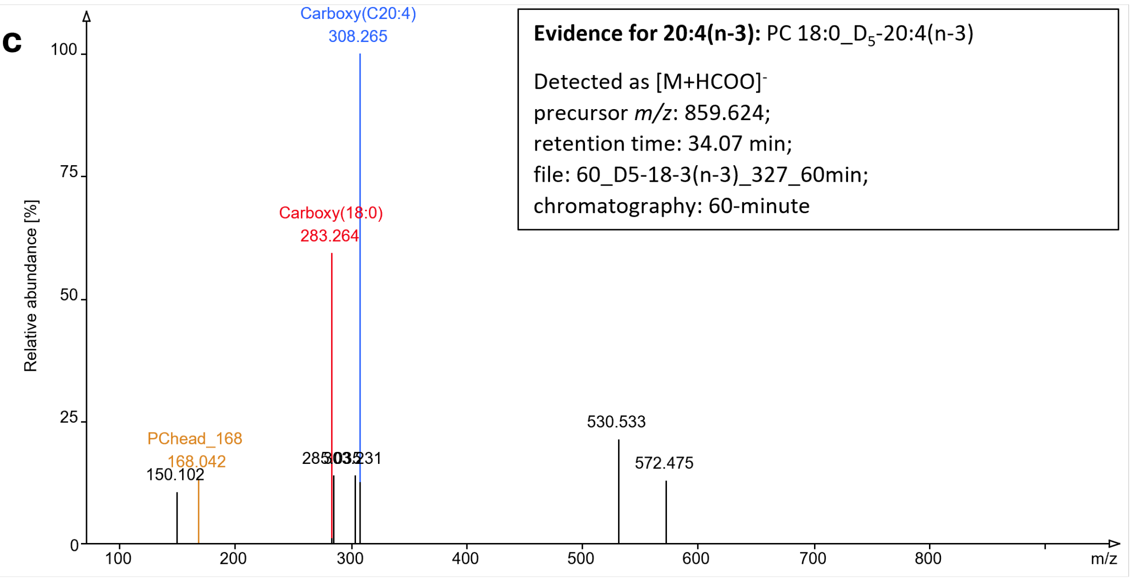

Relative abundance [%]
m/z
Carboxy(C20:4)
308.265
Carboxy(18:0)
283.264
PChead_168
168.042
150.102
530.533
572.475
Evidence for 20:4(n-3): PC 18:0_D5-20:4(n-3)
Detected as [M+HCOO]-
precursor m/z: 859.624;
retention time: 34.07 min;
file: 60_D5-18-3(n-3)_327_60min;
chromatography: 60-minute

ad

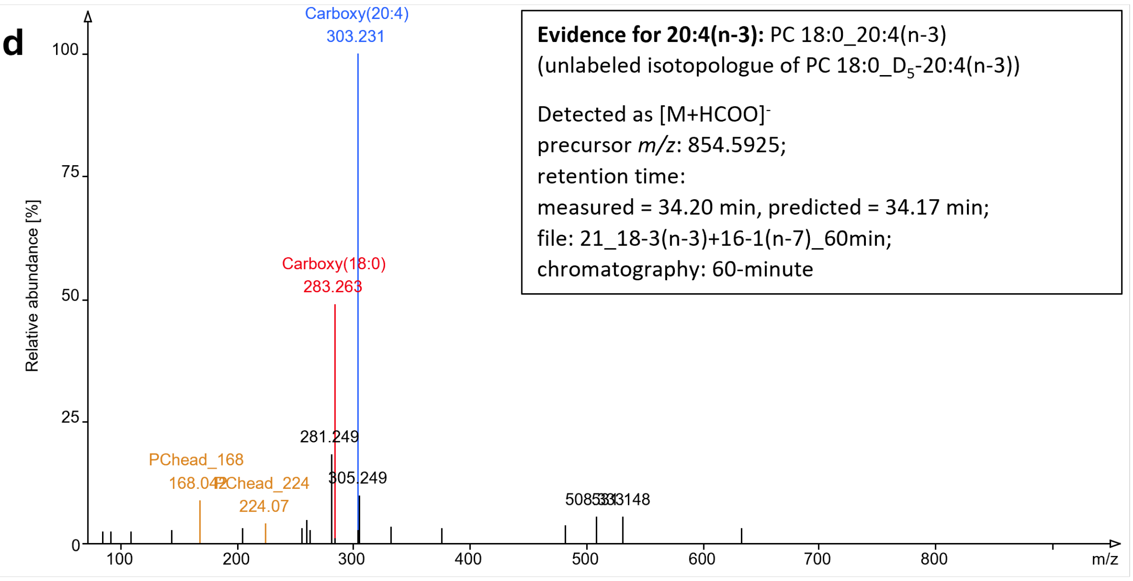

Relative abundance [%]
m/z
Carboxy(20:4)
303.231
Carboxy(18:0)
283.263
281.249
305.249
PChead_168
PChead_224
224.07
Evidence for 20:4(n-3): PC 18:0_20:4(n-3)
(unlabeled isotopologue of PC 18:0_D5-20:4(n-3))
Detected as [M+HCOO]-
precursor m/z: 854.5925;
retention time:
measured = 34.20 min, predicted = 34.17 min;
file: 21_18-3(n-3)+16-1(n-7)_60min;
chromatography: 60-minute

ae

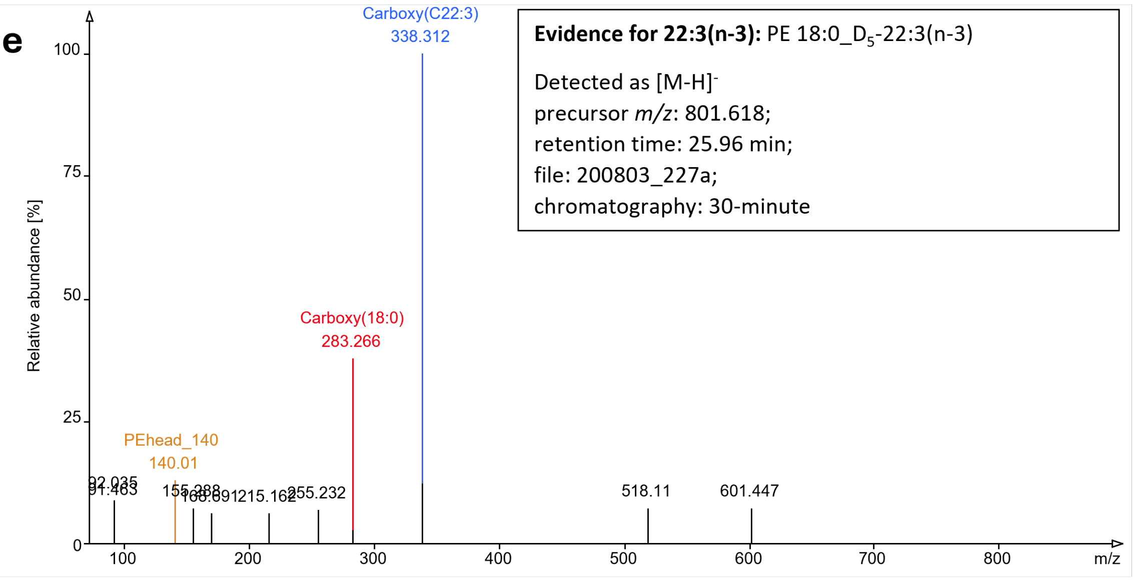

Relative abundance [%]
m/z
Carboxy(C22:3)
338.312
Carboxy(18:0)
283.266
PEhead_140
140.01
255.232
518.11
601.447
Evidence for 22:3(n-3): PE 18:0_D5-22:3(n-3)
Detected as [M-H]-
precursor m/z: 801.618;
retention time: 25.96 min;
file: 200803_227a;
chromatography: 30-minute

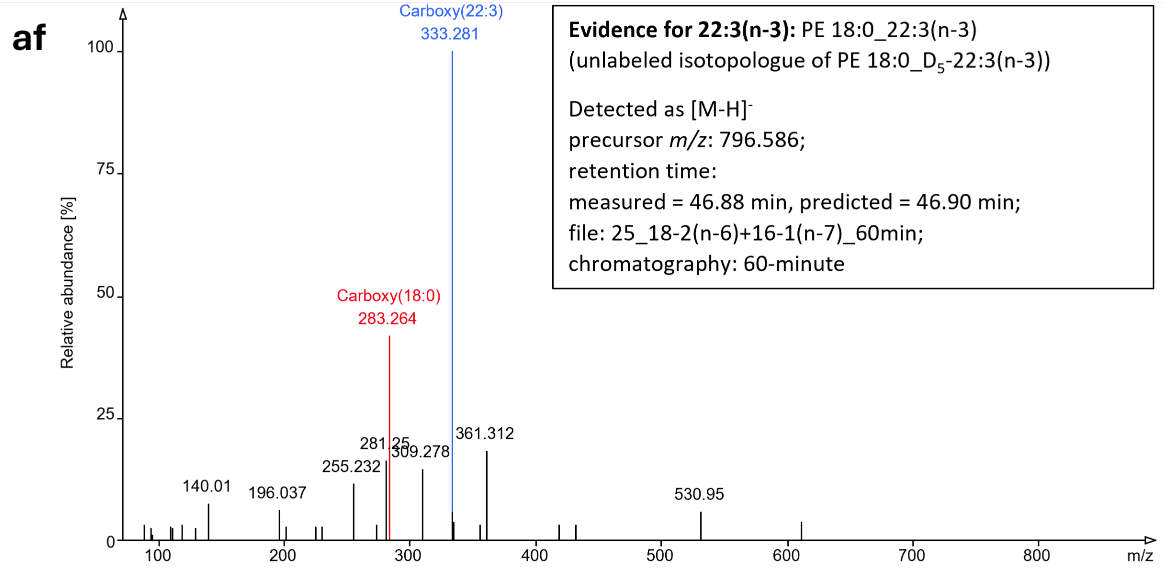
af
Relative abundance [%]
m/z
Carboxy(22:3)
333.281
Carboxy(18:0)
283.264
361.312
309.278
255.232
140.01
196.037
530.95
Evidence for 22:3(n-3): PE 18:0_22:3(n-3)
(unlabeled isotopologue of PE 18:0_D5-22:3(n-3))
Detected as [M-H]-
precursor m/z: 796.586;
retention time:
measured = 46.88 min, predicted = 46.90 min;
file: 25_18-2(n-6)+16-1(n-7)_60min;
chromatography: 60-minute

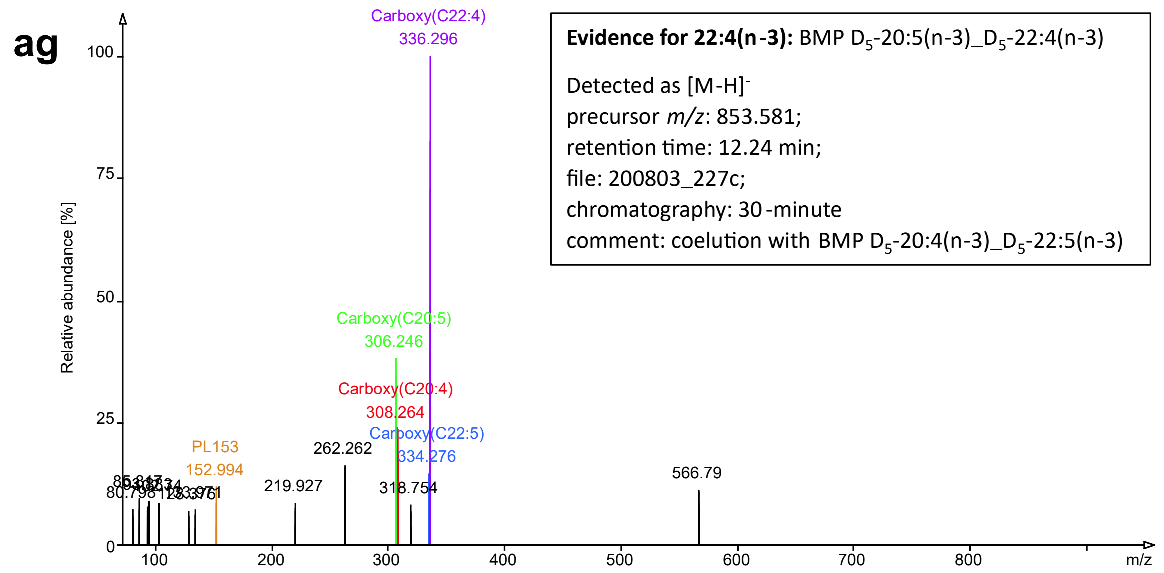
ag
Relative abundance [%]
m/z
Carboxy(C22:4)
336.296
306.246
308.264
334.276
PL153
152.994
262.262
219.927
566.79
Evidence for 22:4(n-3): BMP D5-20:5(n-3)_D5-22:4(n-3)
Detected as [M-H]-
precursor m/z: 853.581;
retention time: 12.24 min;
file: 200803_227c;
chromatography: 30-minute
comment: coelution with BMP D5-20:4(n-3)_D5-22:5(n-3)

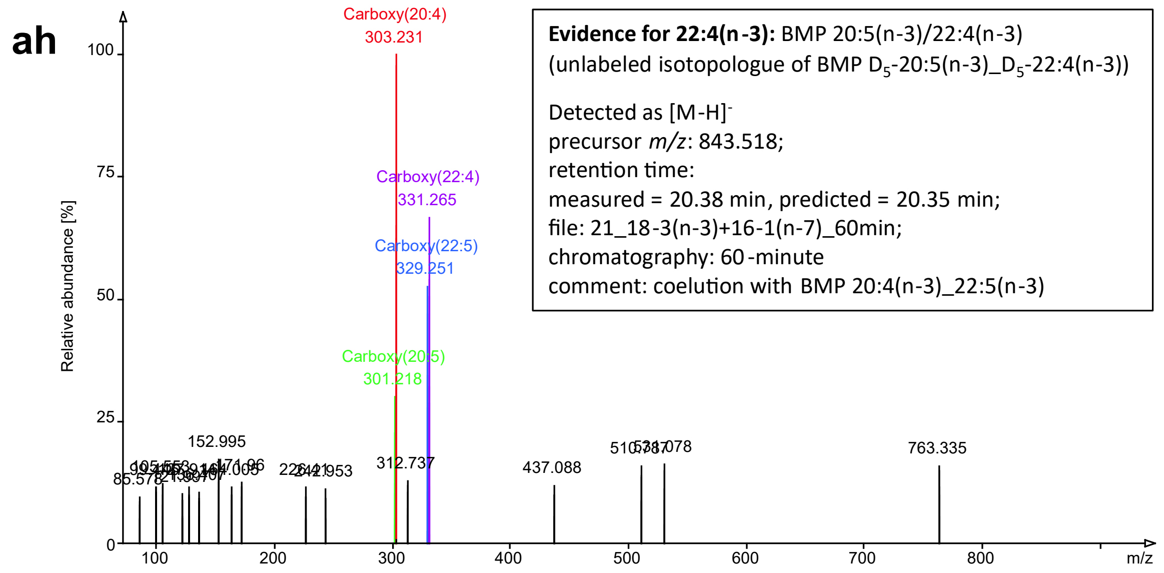
ah
Relative abundance [%]
m/z
Carboxy(20:4)
303.231
331.265
329.251
301.218
152.995
437.088
763.335
Evidence for 22:4(n-3): BMP 20:5(n-3)/22:4(n-3)
(unlabeled isotopologue of BMP D5-20:5(n-3)_D5-22:4(n-3))
Detected as [M-H]-
precursor m/z: 843.518;
retention time:
measured = 20.38 min, predicted = 20.35 min;
file: 21_18-3(n-3)+16-1(n-7)_60min;
chromatography: 60-minute
comment: coelution with BMP 20:4(n-3)_22:5(n-3)

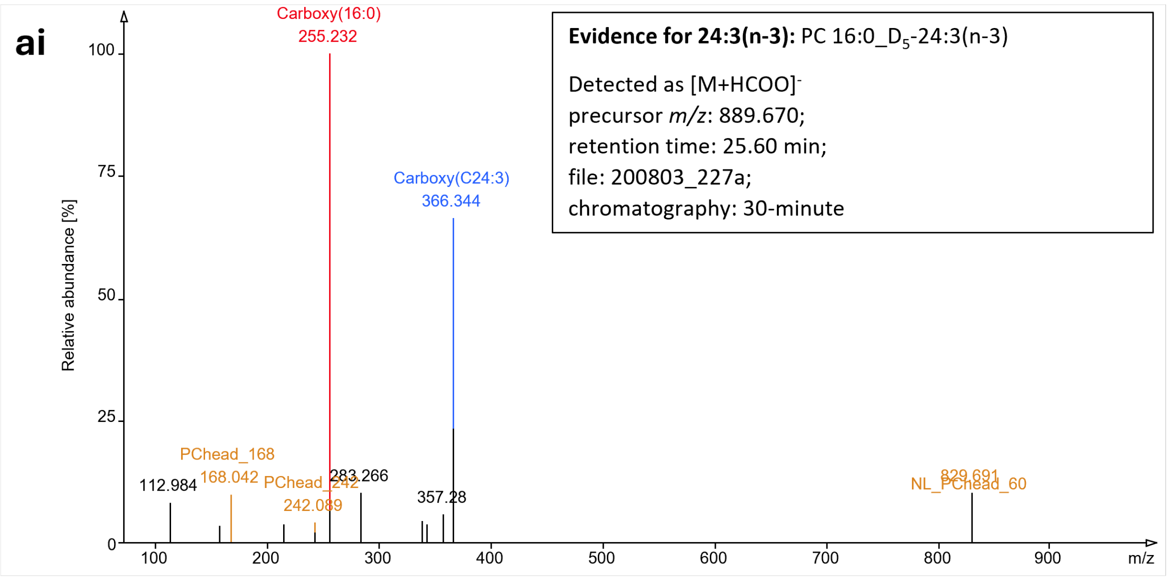

ai
Carboxy(16:0)
255.232
Carboxy(C24:3)
366.344
PCHead_168
168.042
PChead_242
242.089
112.984
283.266
357.28
829.691
NL_PChead_60
Relative abundance [%]
m/z
Evidence for 24:3(n-3): PC 16:0_D5-24:3(n-3)
Detected as [M+HCOO]-
precursor m/z: 889.670;
retention time: 25.60 min;
file: 200803_227a;
chromatography: 30-minute

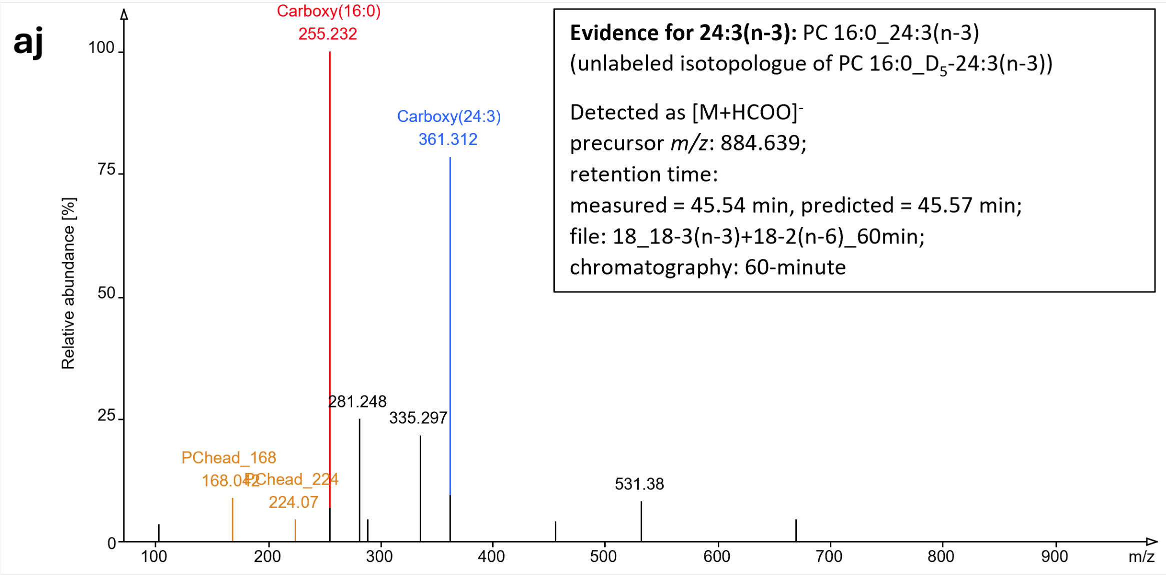

aj
Carboxy(16:0)
255.232
Carboxy(24:3)
361.312
PChead_168
168.042
PChead_224
224.07
281.248
335.297
531.38
Relative abundance [%]
m/z
Evidence for 24:3(n-3): PC 16:0_24:3(n-3)
(unlabeled isotopologue of PC 16:0_D5-24:3(n-3))
Detected as [M+HCOO]-
precursor m/z: 884.639;
retention time:
measured = 45.54 min, predicted = 45.57 min;
file: 18_18-3(n-3)+18-2(n-6)_60min;
chromatography: 60-minute

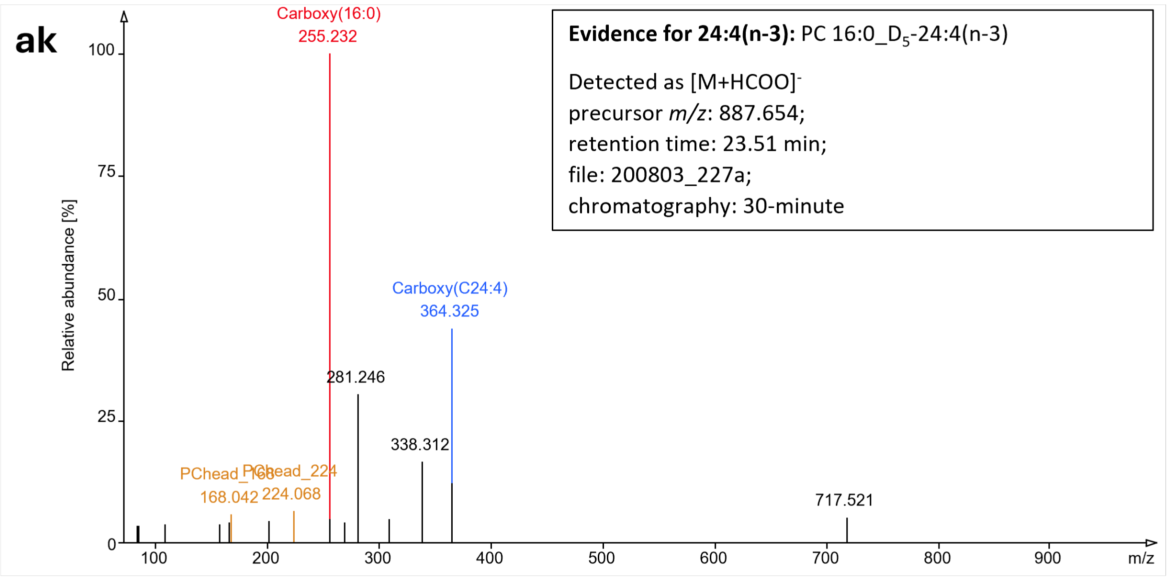

ak
Carboxy(16:0)
255.232
Carboxy(C24:4)
364.325
PChead_168
168.042
PChead_224
224.068
281.246
338.312
717.521
Relative abundance [%]
m/z
Evidence for 24:4(n-3): PC 16:0_D5-24:4(n-3)
Detected as [M+HCOO]-
precursor m/z: 887.654;
retention time: 23.51 min;
file: 200803_227a;
chromatography: 30-minute

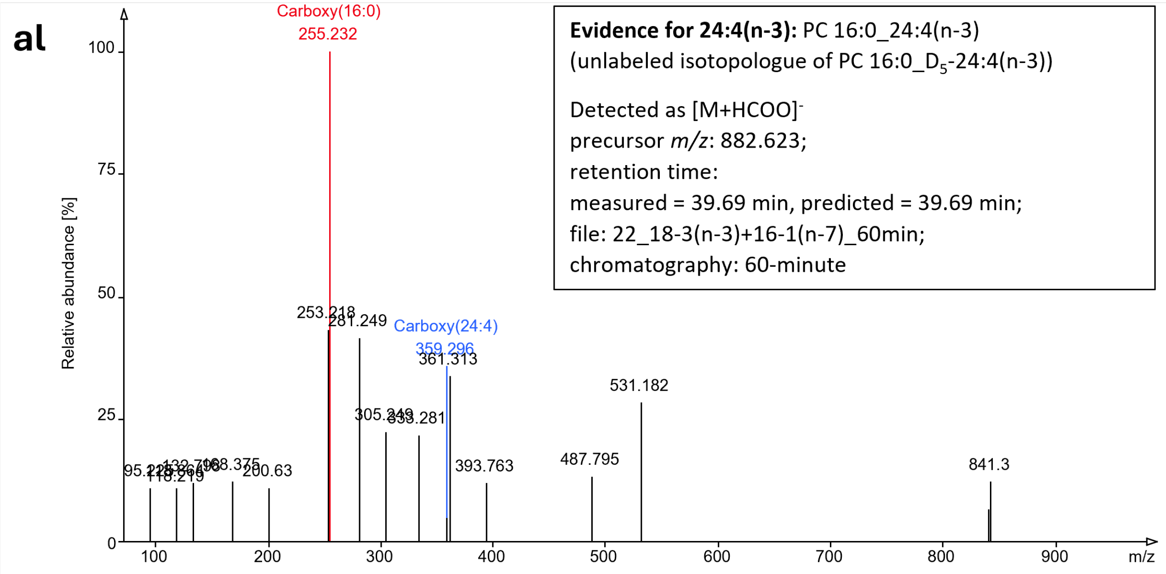
al
Carboxy(16:0)
255.232
253.218
281.249
Carboxy(24:4)
359.296
361.313
333.281
393.763
487.795
531.182
841.3
200.63
Relative abundance [%]
m/z
Evidence for 24:4(n-3): PC 16:0_24:4(n-3)
(unlabeled isotopologue of PC 16:0_D$_5$-24:4(n-3))
Detected as [M+HCOO]$^-$
precursor m/z: 882.623;
retention time:
measured = 39.69 min, predicted = 39.69 min;
file: 22_18-3(n-3)+16-1(n-7)_60min;
chromatography: 60-minute

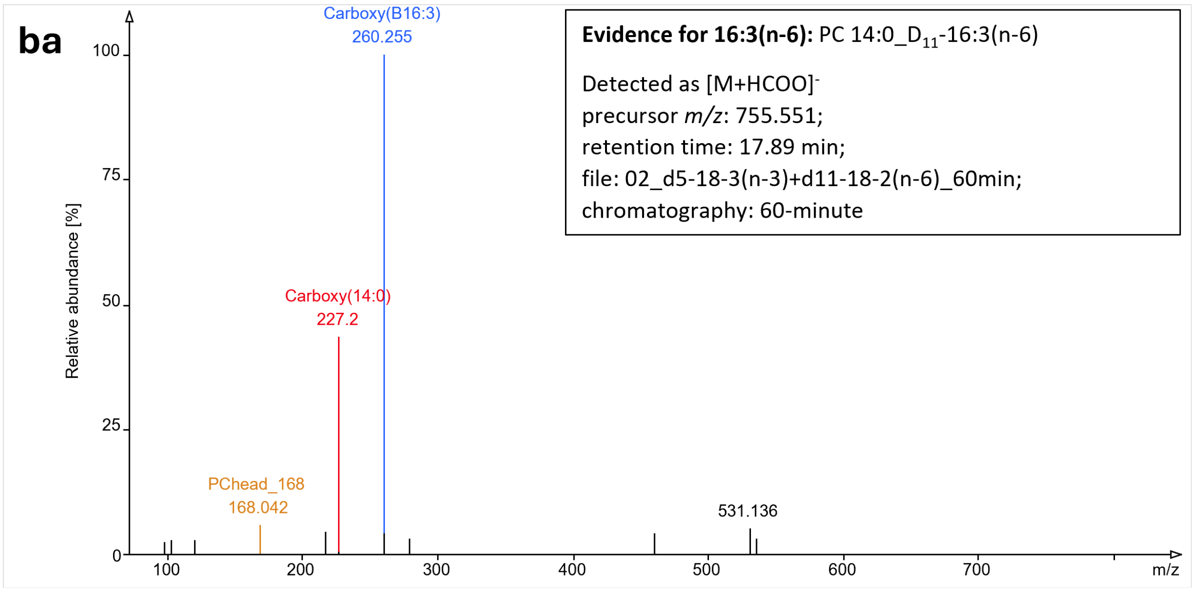
ba
Carboxy(B16:3)
260.255
Carboxy(14:0)
227.2
PChead_168
168.042
531.136
Relative abundance [%]
m/z
Evidence for 16:3(n-6): PC 14:0_D$_{11}$-16:3(n-6)
Detected as [M+HCOO]$^-$
precursor m/z: 755.551;
retention time: 17.89 min;
file: 02_d5-18-3(n-3)+d11-18-2(n-6)_60min;
chromatography: 60-minute

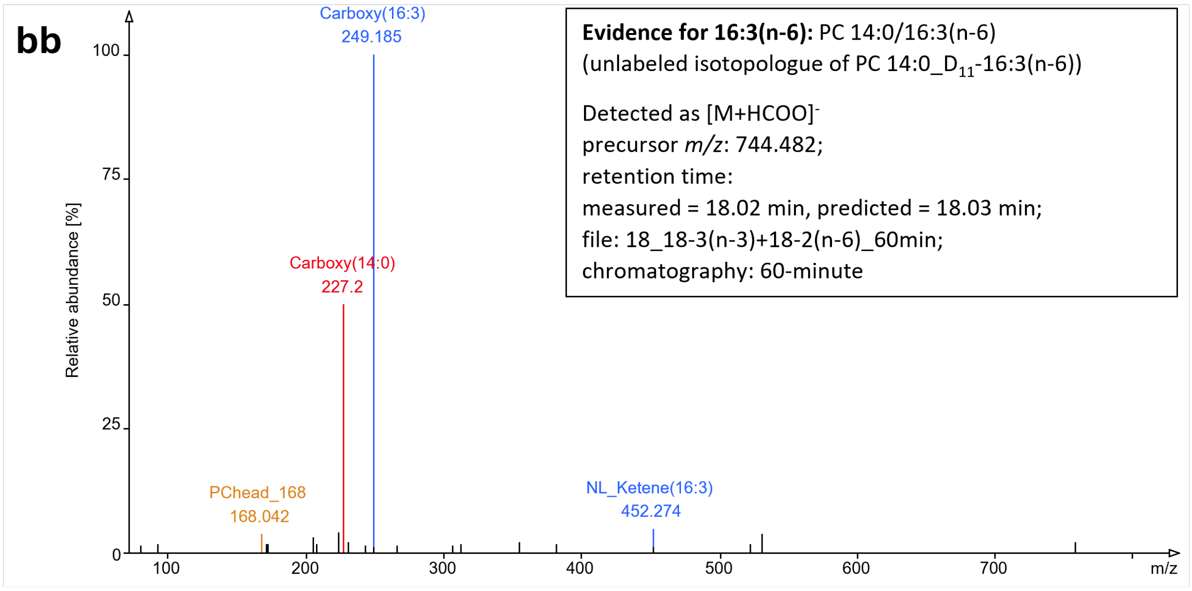
bb
Carboxy(16:3)
249.185
Carboxy(14:0)
227.2
PChead_168
168.042
NL_Ketene(16:3)
452.274
Relative abundance [%]
m/z
Evidence for 16:3(n-6): PC 14:0/16:3(n-6)
(unlabeled isotopologue of PC 14:0_D$_{11}$-16:3(n-6))
Detected as [M+HCOO]$^-$
precursor m/z: 744.482;
retention time:
measured = 18.02 min, predicted = 18.03 min;
file: 18_18-3(n-3)+18-2(n-6)_60min;
chromatography: 60-minute

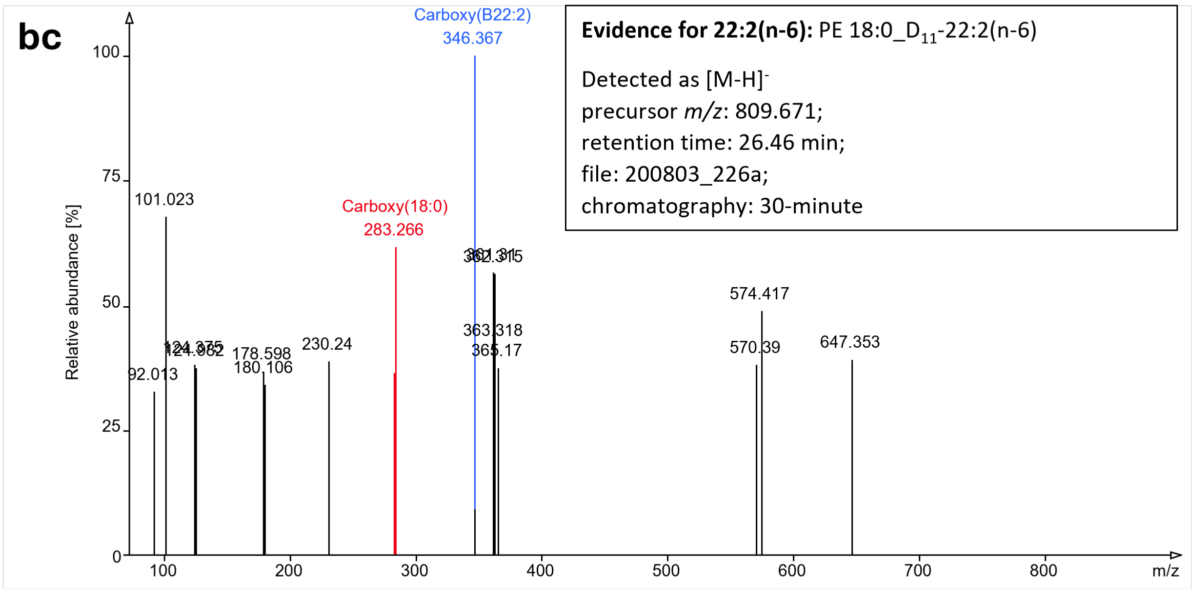
bc
Evidence for 22:2(n-6): PE 18:0_D_{11}-22:2(n-6)
Detected as $[M-H]^-$
precursor *m/z*: 809.671;
retention time: 26.46 min;
file: 200803_226a;
chromatography: 30-minute
Carboxy(B22:2)
346.367
Carboxy(18:0)
283.266
101.023
92.013
178.598
180.106
230.24
363.318
574.417
570.39
647.353
Relative abundance [%]
m/z

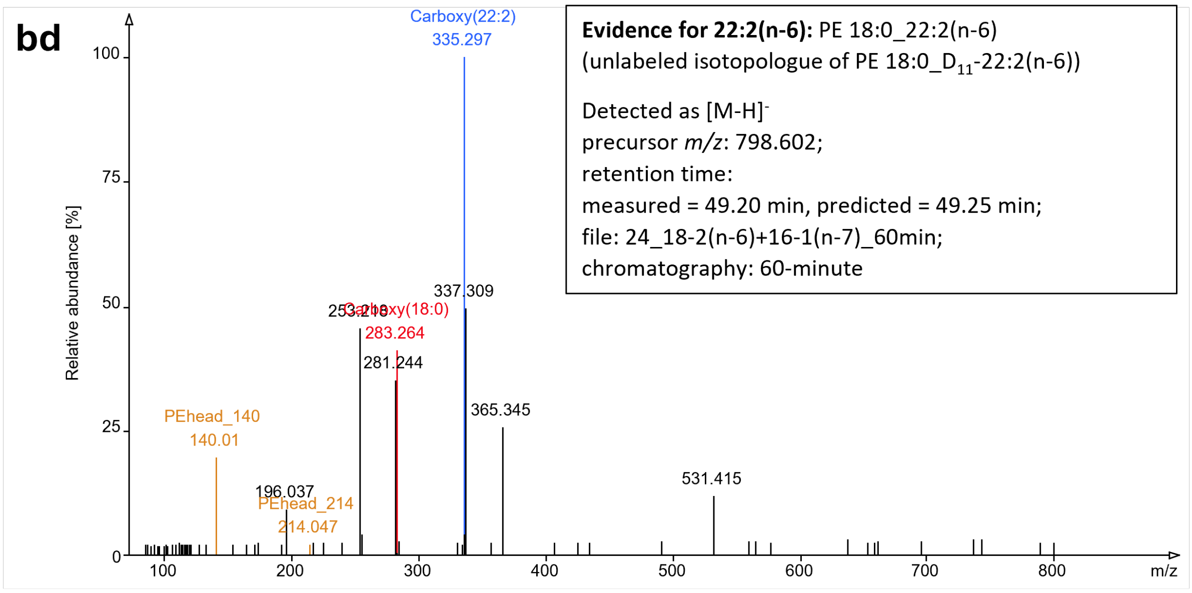
bd
Evidence for 22:2(n-6): PE 18:0_22:2(n-6)
(unlabeled isotopologue of PE 18:0_D_{11}-22:2(n-6))
Detected as $[M-H]^-$
precursor *m/z*: 798.602;
retention time:
measured = 49.20 min, predicted = 49.25 min;
file: 24_18-2(n-6)+16-1(n-7)_60min;
chromatography: 60-minute
Carboxy(22:2)
335.297
283.264
281.244
337.309
365.345
PEhead_140
140.01
196.037
214.047
531.415
Relative abundance [%]
m/z

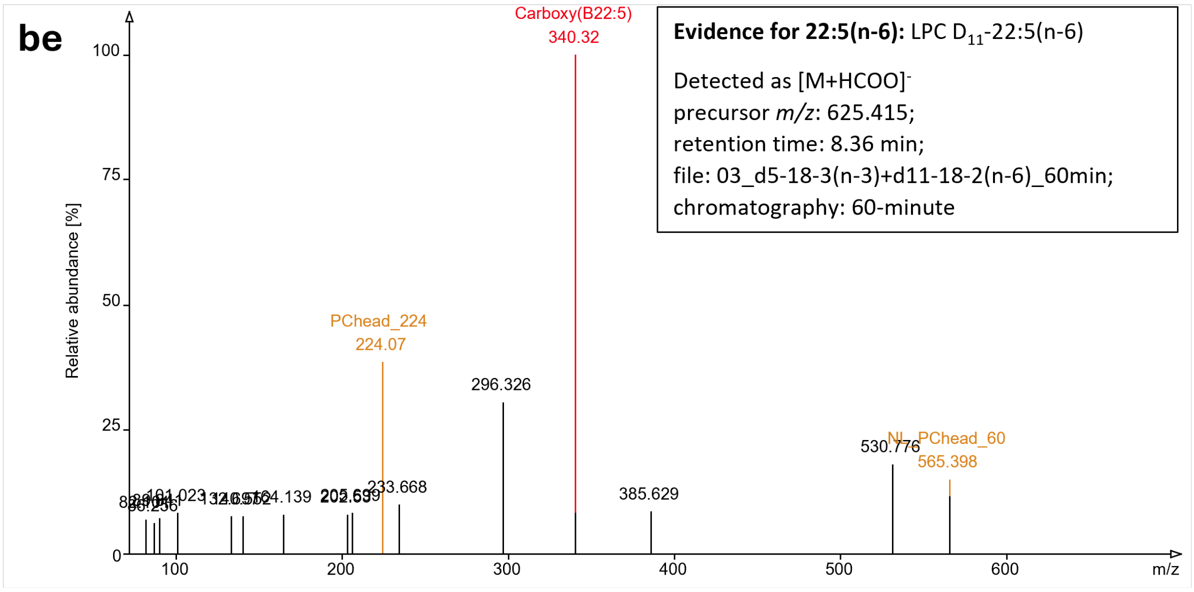
be
Evidence for 22:5(n-6): LPC D_{11}-22:5(n-6)
Detected as $[M+HCOO]^-$
precursor *m/z*: 625.415;
retention time: 8.36 min;
file: 03_d5-18-3(n-3)+d11-18-2(n-6)_60min;
chromatography: 60-minute
Carboxy(B22:5)
340.32
PChead_224
224.07
296.326
233.668
385.629
530.776
565.398
Relative abundance [%]
m/z

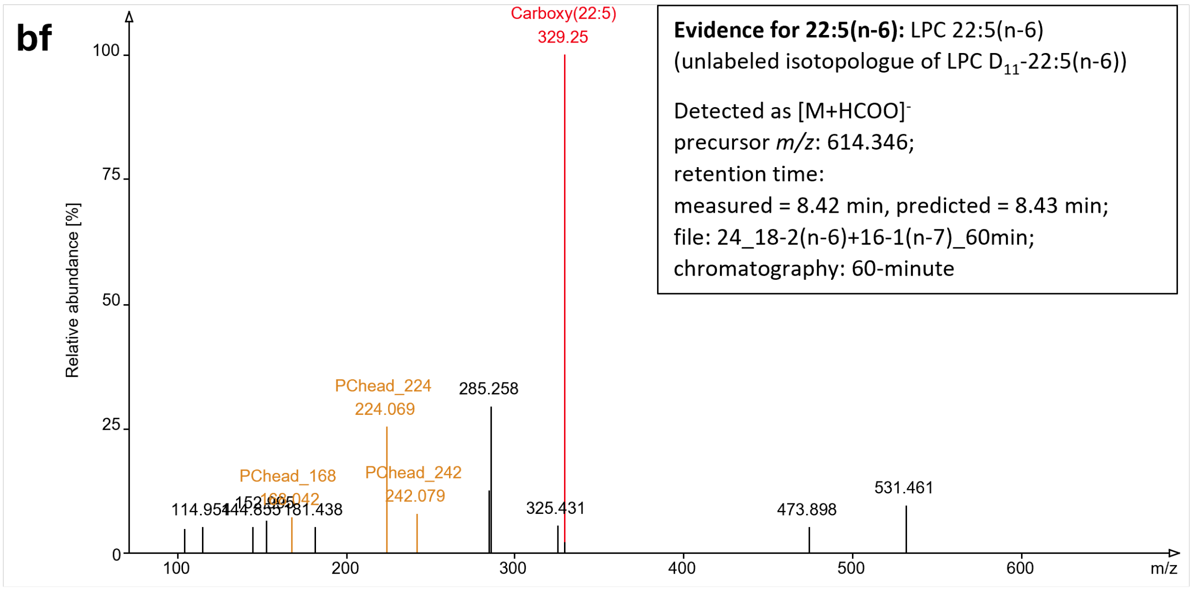
bf
Relative abundance [%]
m/z
Carboxy(22:5)
329.25
PChead_224
224.069
285.258
PChead_168
PChead_242
242.079
114.954
181.438
325.431
473.898
531.461
Evidence for 22:5(n-6): LPC 22:5(n-6)
(unlabeled isotopologue of LPC D_{11}-22:5(n-6))
Detected as $[M+HCOO]^-$
precursor *m/z*: 614.346;
retention time:
measured = 8.42 min, predicted = 8.43 min;
file: 24_18-2(n-6)+16-1(n-7)_60min;
chromatography: 60-minute

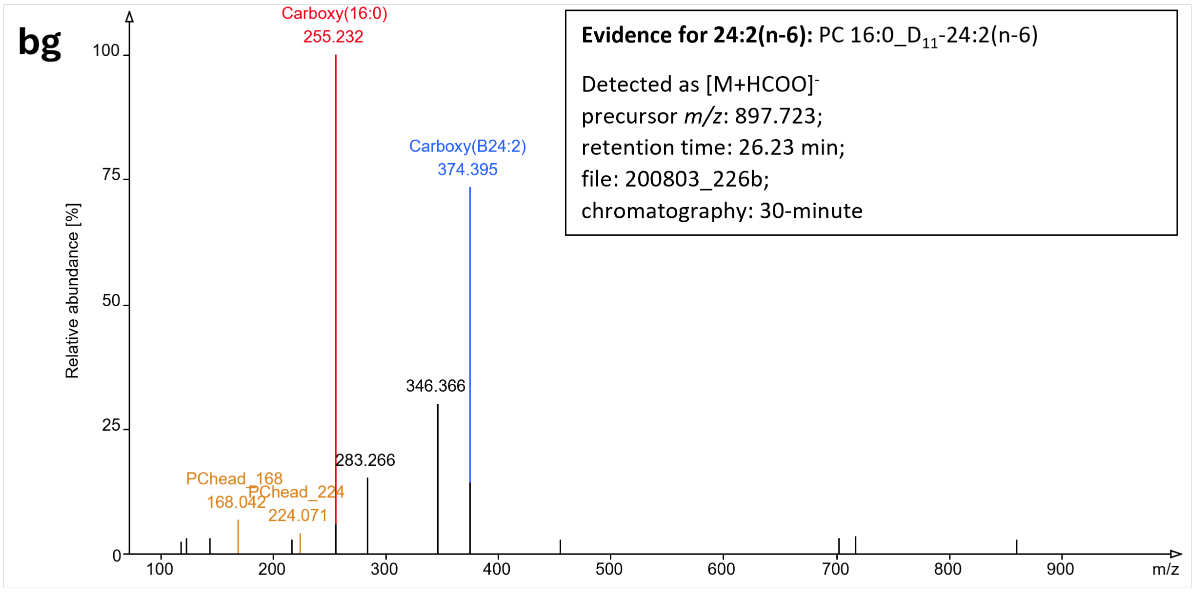
bg
Relative abundance [%]
m/z
Carboxy(16:0)
255.232
Carboxy(B24:2)
374.395
346.366
283.266
PChead_168
168.042
PChead_224
224.071
Evidence for 24:2(n-6): PC 16:0_D_{11}-24:2(n-6)
Detected as $[M+HCOO]^-$
precursor *m/z*: 897.723;
retention time: 26.23 min;
file: 200803_226b;
chromatography: 30-minute

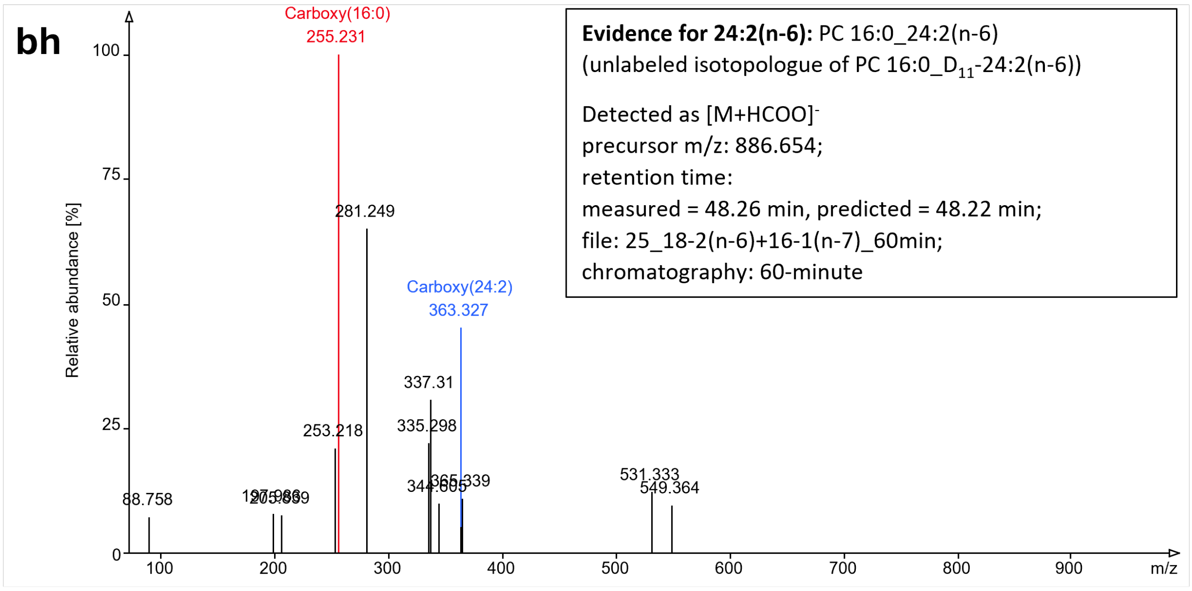
bh
Relative abundance [%]
m/z
Carboxy(16:0)
255.231
281.249
Carboxy(24:2)
363.327
337.31
335.298
253.218
88.758
531.333
549.364
Evidence for 24:2(n-6): PC 16:0_24:2(n-6)
(unlabeled isotopologue of PC 16:0_D_{11}-24:2(n-6))
Detected as $[M+HCOO]^-$
precursor m/z: 886.654;
retention time:
measured = 48.26 min, predicted = 48.22 min;
file: 25_18-2(n-6)+16-1(n-7)_60min;
chromatography: 60-minute

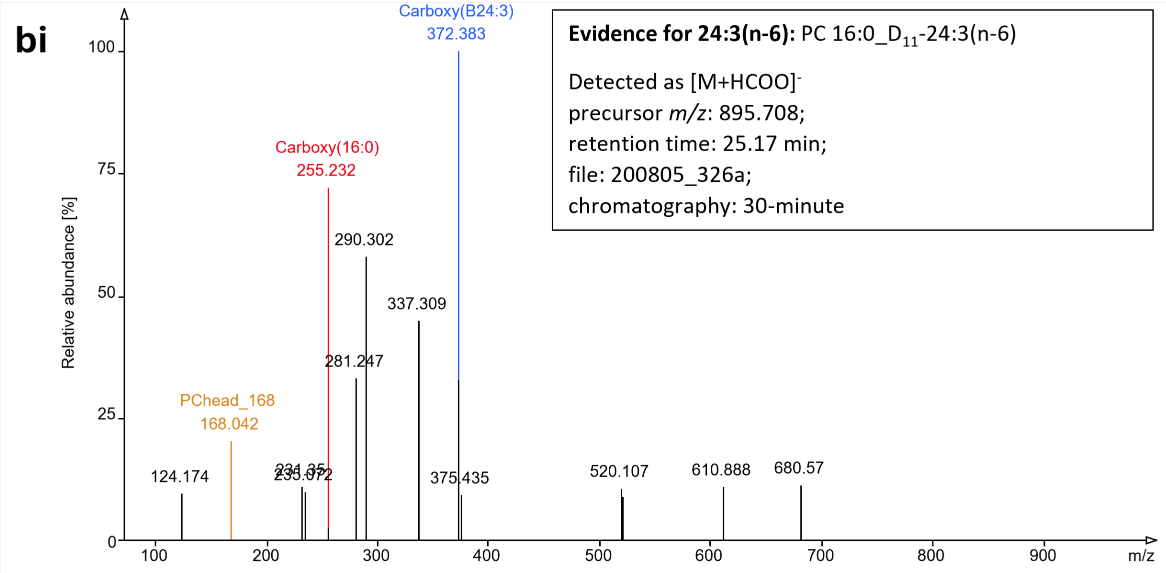
bi
Carboxy(B24:3)
372.383
Carboxy(16:0)
255.232
290.302
337.309
281.247
PChead_168
168.042
124.174
375.435
520.107
610.888
680.57
Relative abundance [%]
m/z
Evidence for 24:3(n-6): PC 16:0_D$_{11}$-24:3(n-6)
Detected as [M+HCOO]$^-$
precursor *m/z*: 895.708;
retention time: 25.17 min;
file: 200805_326a;
chromatography: 30-minute

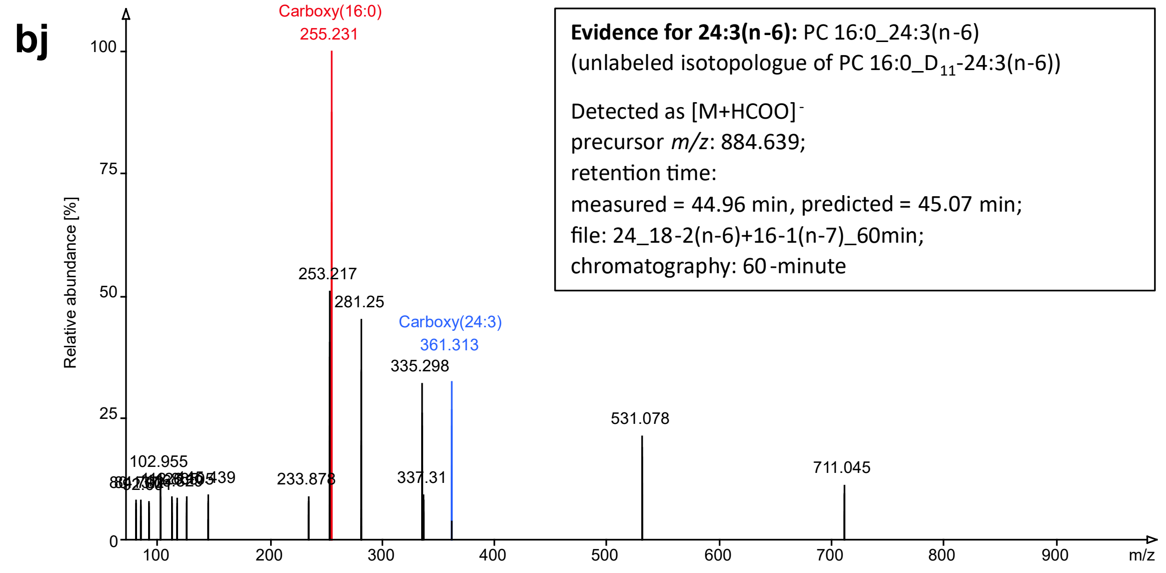
bj
Carboxy(16:0)
255.231
253.217
281.25
Carboxy(24:3)
361.313
335.298
531.078
711.045
102.955
233.878
337.31
Relative abundance [%]
m/z
Evidence for 24:3(n-6): PC 16:0_24:3(n-6)
(unlabeled isotopologue of PC 16:0_D$_{11}$-24:3(n-6))
Detected as [M+HCOO]$^-$
precursor *m/z*: 884.639;
retention time:
measured = 44.96 min, predicted = 45.07 min;
file: 24_18-2(n-6)+16-1(n-7)_60min;
chromatography: 60-minute

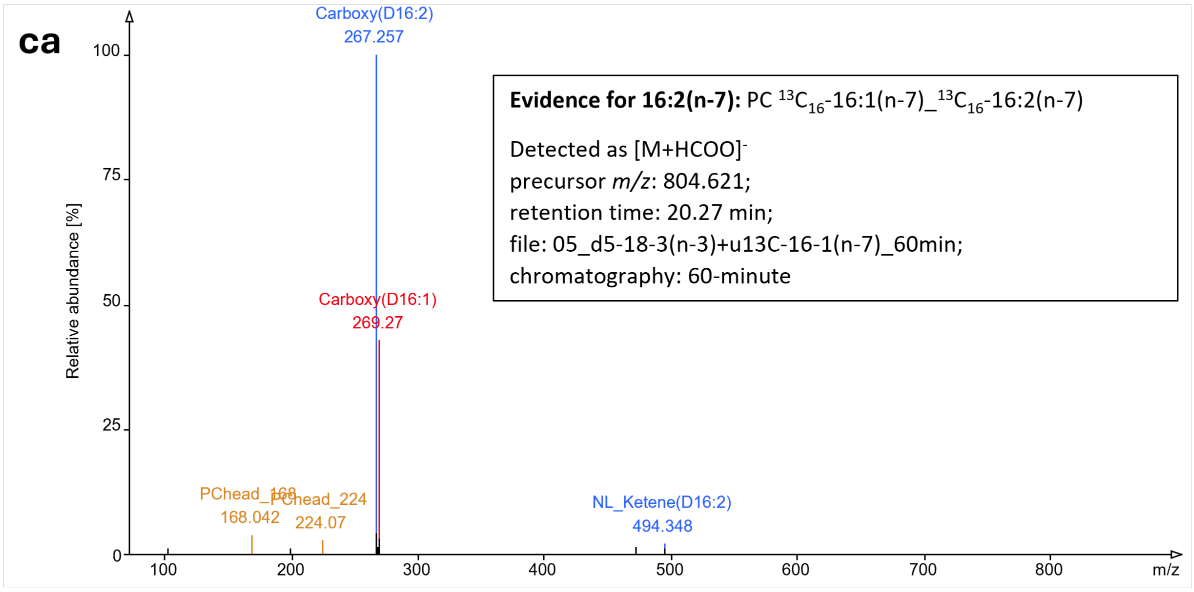
ca
Carboxy(D16:2)
267.257
Carboxy(D16:1)
269.27
168.042
224.07
NL_Ketene(D16:2)
494.348
Relative abundance [%]
m/z
Evidence for 16:2(n-7): PC $^{13}C_{16}$-16:1(n-7)_$^{13}C_{16}$-16:2(n-7)
Detected as [M+HCOO]$^-$
precursor *m/z*: 804.621;
retention time: 20.27 min;
file: 05_d5-18-3(n-3)+u13C-16-1(n-7)_60min;
chromatography: 60-minute

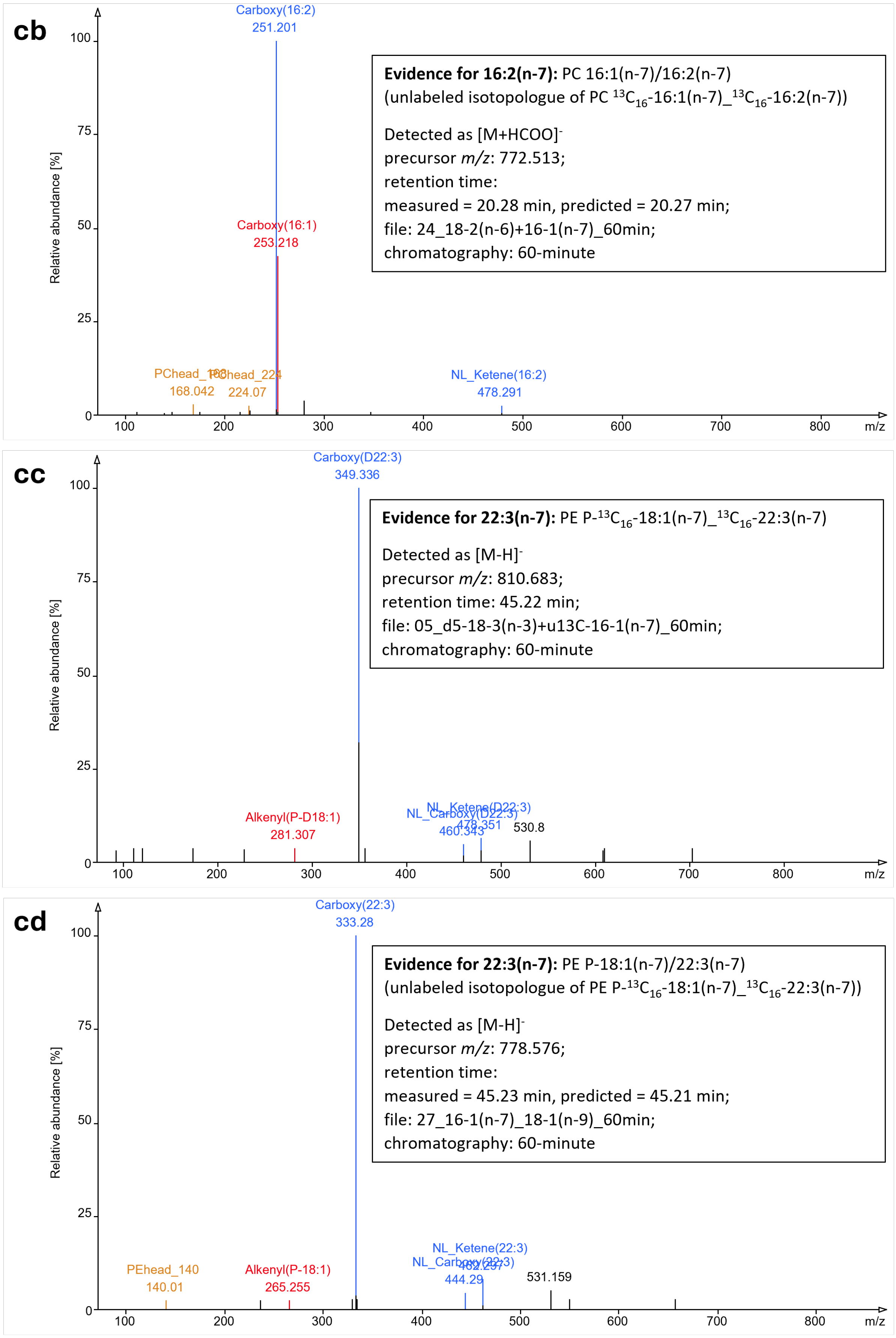

cb
Relative abundance [%]
Carboxy(16:2)
251.201
Carboxy(16:1)
253.218
168.042
224.07
NL_Ketene(16:2)
478.291
m/z
Evidence for 16:2(n-7): PC 16:1(n-7)/16:2(n-7)
(unlabeled isotopologue of PC $^{13}C_{16}$-16:1(n-7)_$^{13}C_{16}$-16:2(n-7))
Detected as $[M+HCOO]^-$
precursor *m/z*: 772.513;
retention time:
measured = 20.28 min, predicted = 20.27 min;
file: 24_18-2(n-6)+16-1(n-7)_60min;
chromatography: 60-minute
cc
Relative abundance [%]
Carboxy(D22:3)
349.336
Alkenyl(P-D18:1)
281.307
530.8
m/z
Evidence for 22:3(n-7): PE P-$^{13}C_{16}$-18:1(n-7)_$^{13}C_{16}$-22:3(n-7)
Detected as $[M-H]^-$
precursor *m/z*: 810.683;
retention time: 45.22 min;
file: 05_d5-18-3(n-3)+u13C-16-1(n-7)_60min;
chromatography: 60-minute
cd
Relative abundance [%]
Carboxy(22:3)
333.28
PEhead_140
140.01
Alkenyl(P-18:1)
265.255
NL_Ketene(22:3)
444.29
531.159
m/z
Evidence for 22:3(n-7): PE P-18:1(n-7)/22:3(n-7)
(unlabeled isotopologue of PE P-$^{13}C_{16}$-18:1(n-7)_$^{13}C_{16}$-22:3(n-7))
Detected as $[M-H]^-$
precursor *m/z*: 778.576;
retention time:
measured = 45.23 min, predicted = 45.21 min;
file: 27_16-1(n-7)_18-1(n-9)_60min;
chromatography: 60-minute

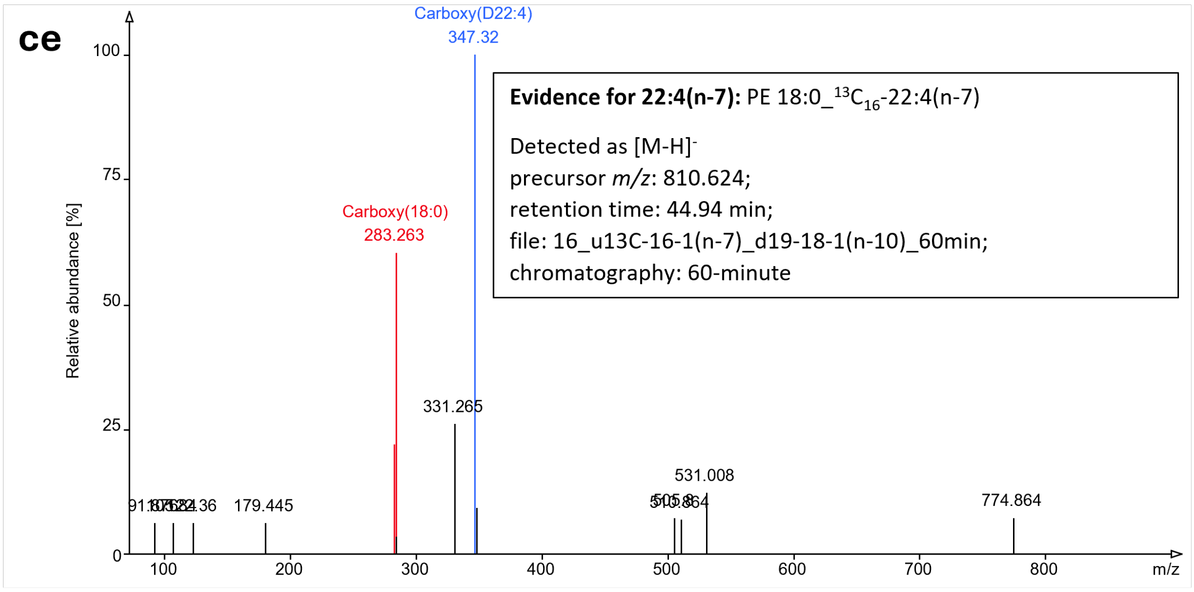
ce
Carboxy(D22:4)
347.32
Carboxy(18:0)
283.263
331.265
531.008
774.864
179.445
Relative abundance [%]
m/z
Evidence for 22:4(n-7): PE 18:0_$^{13}C_{16}$-22:4(n-7)
Detected as $[M-H]^-$
precursor m/z: 810.624;
retention time: 44.94 min;
file: 16_u13C-16-1(n-7)_d19-18-1(n-10)_60min;
chromatography: 60-minute

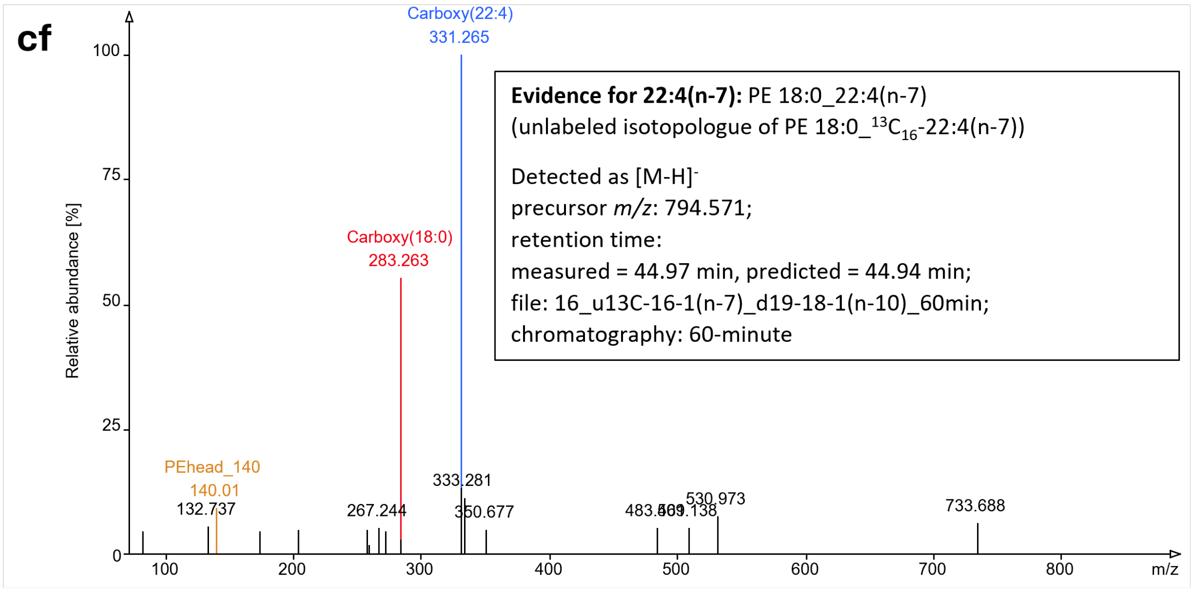
cf
Carboxy(22:4)
331.265
Carboxy(18:0)
283.263
PEhead_140
140.01
132.737
267.244
333.281
350.677
530.973
733.688
Relative abundance [%]
m/z
Evidence for 22:4(n-7): PE 18:0_22:4(n-7)
(unlabeled isotopologue of PE 18:0_$^{13}C_{16}$-22:4(n-7))
Detected as $[M-H]^-$
precursor m/z: 794.571;
retention time:
measured = 44.97 min, predicted = 44.94 min;
file: 16_u13C-16-1(n-7)_d19-18-1(n-10)_60min;
chromatography: 60-minute

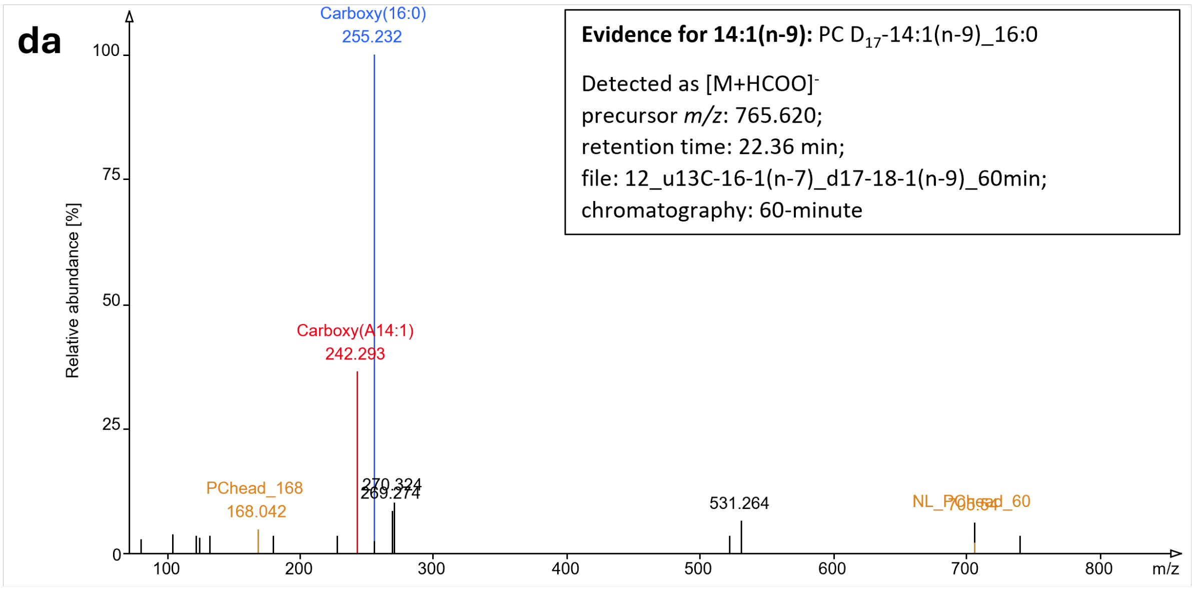
da
Carboxy(16:0)
255.232
Carboxy(A14:1)
242.293
PChead_168
168.042
531.264
Relative abundance [%]
m/z
Evidence for 14:1(n-9): PC D_{17}-14:1(n-9)_16:0
Detected as $[M+HCOO]^-$
precursor m/z: 765.620;
retention time: 22.36 min;
file: 12_u13C-16-1(n-7)_d17-18-1(n-9)_60min;
chromatography: 60-minute

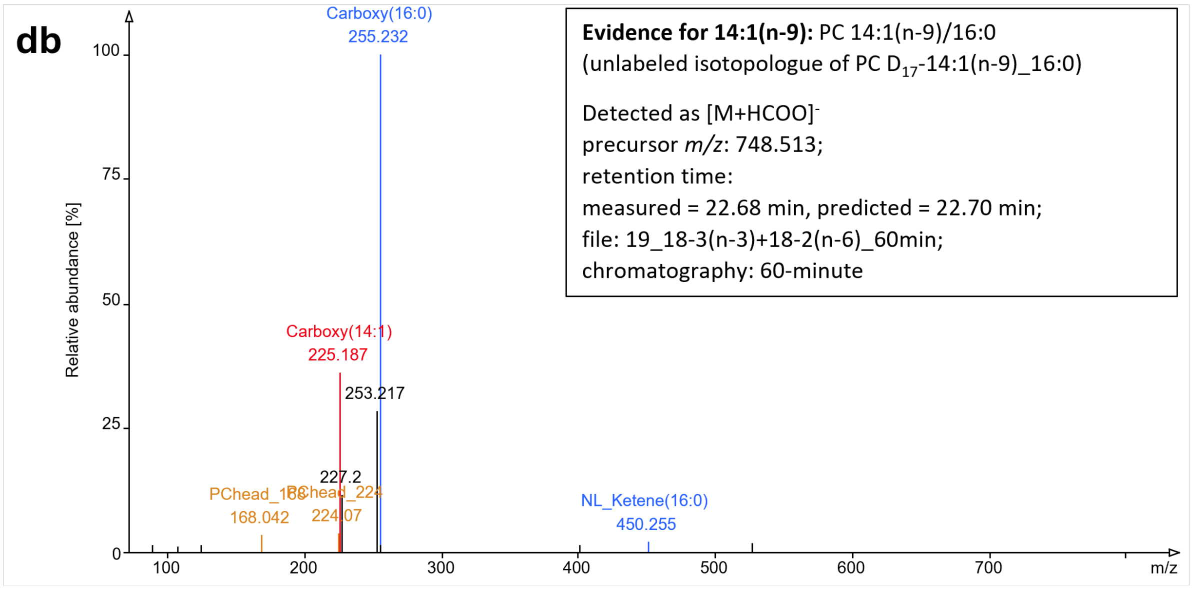
db
Relative abundance [%]
100
75
50
25
0
Carboxy(16:0)
255.232
Carboxy(14:1)
225.187
253.217
227.2
PChead_168
168.042
PChead_224
224.07
NL_Ketene(16:0)
450.255
100
200
300
400
500
600
700
m/z
Evidence for 14:1(n-9): PC 14:1(n-9)/16:0
(unlabeled isotopologue of PC D_{17}-14:1(n-9)_16:0)
Detected as $[M+HCOO]^-$
precursor *m/z*: 748.513;
retention time:
measured = 22.68 min, predicted = 22.70 min;
file: 19_18-3(n-3)+18-2(n-6)_60min;
chromatography: 60-minute

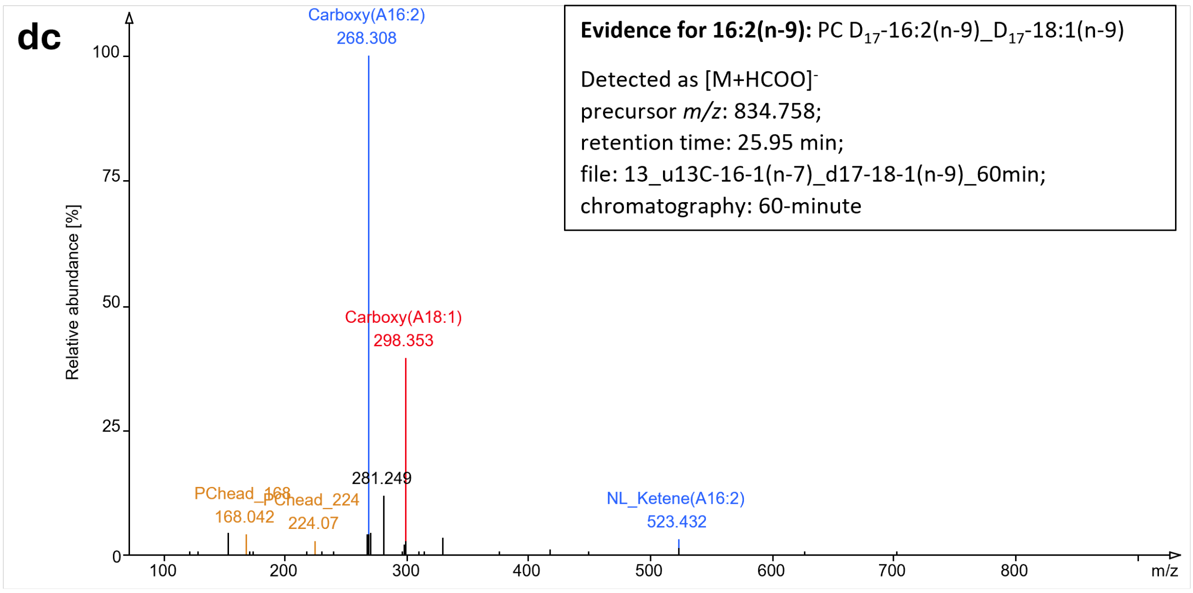
dc
Relative abundance [%]
100
75
50
25
0
Carboxy(A16:2)
268.308
Carboxy(A18:1)
298.353
281.249
PChead_168
168.042
PChead_224
224.07
NL_Ketene(A16:2)
523.432
100
200
300
400
500
600
700
800
m/z
Evidence for 16:2(n-9): PC D_{17}-16:2(n-9)_D_{17}-18:1(n-9)
Detected as $[M+HCOO]^-$
precursor *m/z*: 834.758;
retention time: 25.95 min;
file: 13_u13C-16-1(n-7)_d17-18-1(n-9)_60min;
chromatography: 60-minute

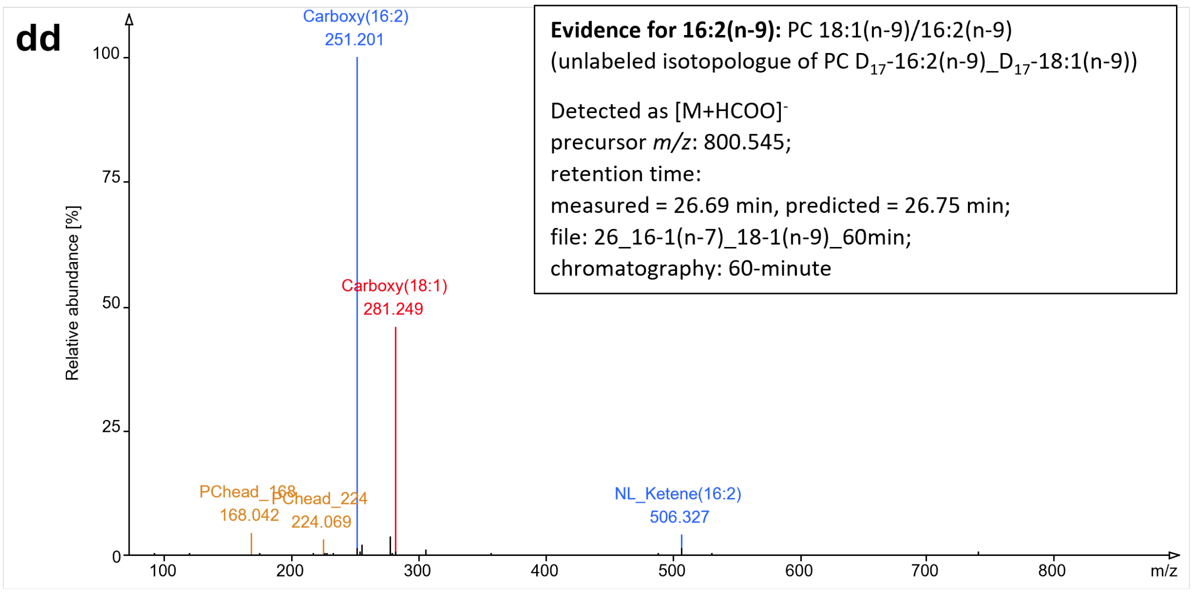
dd
Relative abundance [%]
100
75
50
25
0
Carboxy(16:2)
251.201
Carboxy(18:1)
281.249
PChead_168
168.042
PChead_224
224.069
NL_Ketene(16:2)
506.327
100
200
300
400
500
600
700
800
m/z
Evidence for 16:2(n-9): PC 18:1(n-9)/16:2(n-9)
(unlabeled isotopologue of PC D_{17}-16:2(n-9)_D_{17}-18:1(n-9))
Detected as $[M+HCOO]^-$
precursor *m/z*: 800.545;
retention time:
measured = 26.69 min, predicted = 26.75 min;
file: 26_16-1(n-7)_18-1(n-9)_60min;
chromatography: 60-minute

de

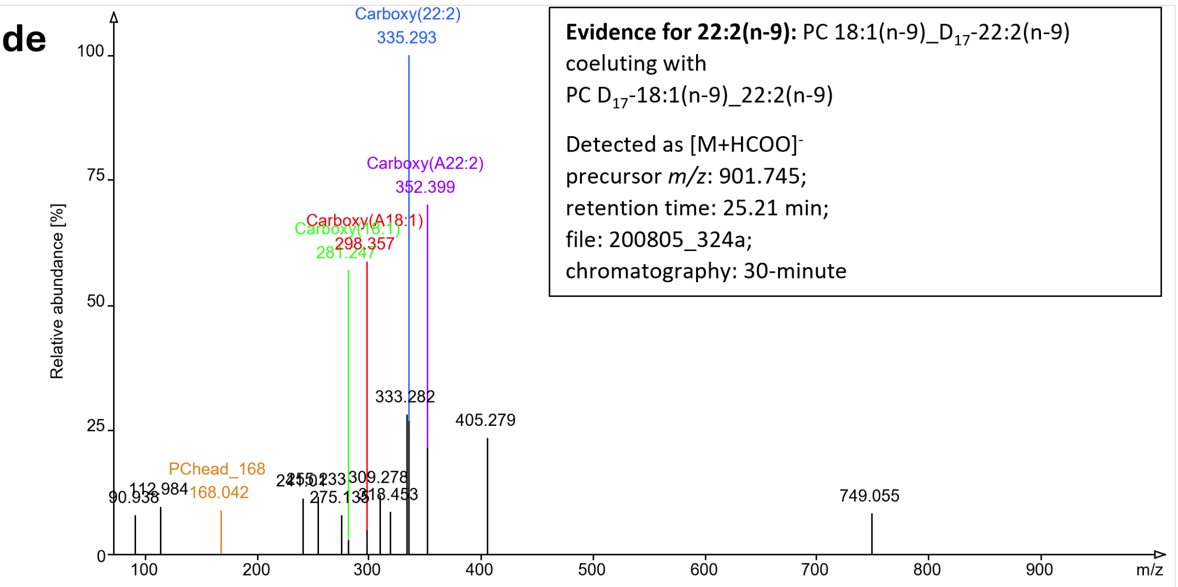

Evidence for 22:2(n-9): PC 18:1(n-9)_D_{17}-22:2(n-9) coeluting with PC D_{17}-18:1(n-9)_22:2(n-9)

Detected as $[M+HCOO]^-$
precursor *m/z*: 901.745;
retention time: 25.21 min;
file: 200805_324a;
chromatography: 30-minute

df

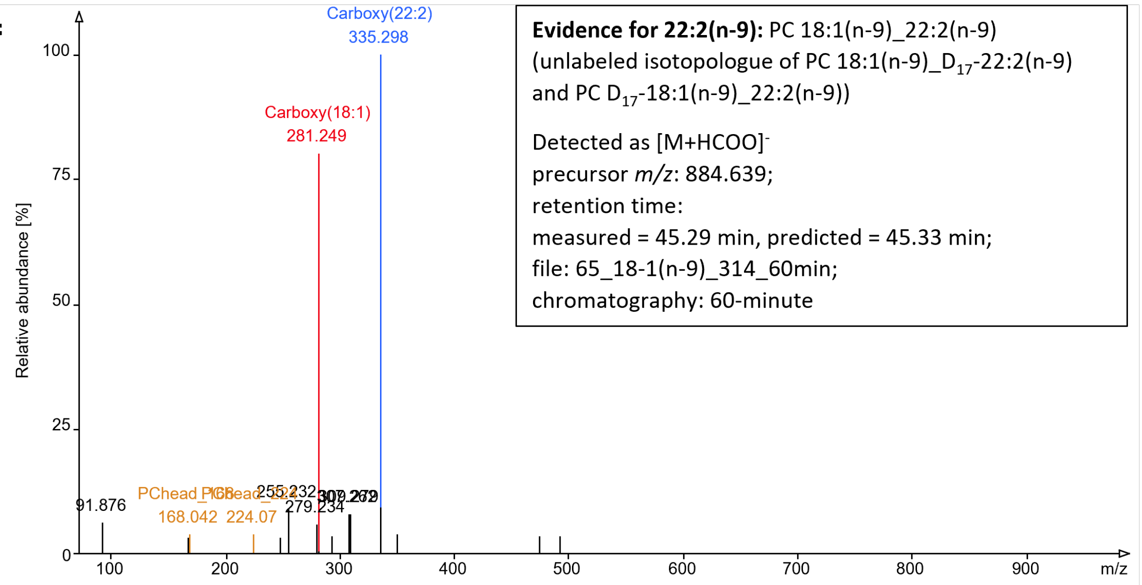

Evidence for 22:2(n-9): PC 18:1(n-9)_22:2(n-9) (unlabeled isotopologue of PC 18:1(n-9)_D_{17}-22:2(n-9) and PC D_{17}-18:1(n-9)_22:2(n-9))

Detected as $[M+HCOO]^-$
precursor *m/z*: 884.639;
retention time:
measured = 45.29 min, predicted = 45.33 min;
file: 65_18-1(n-9)_314_60min;
chromatography: 60-minute

dg

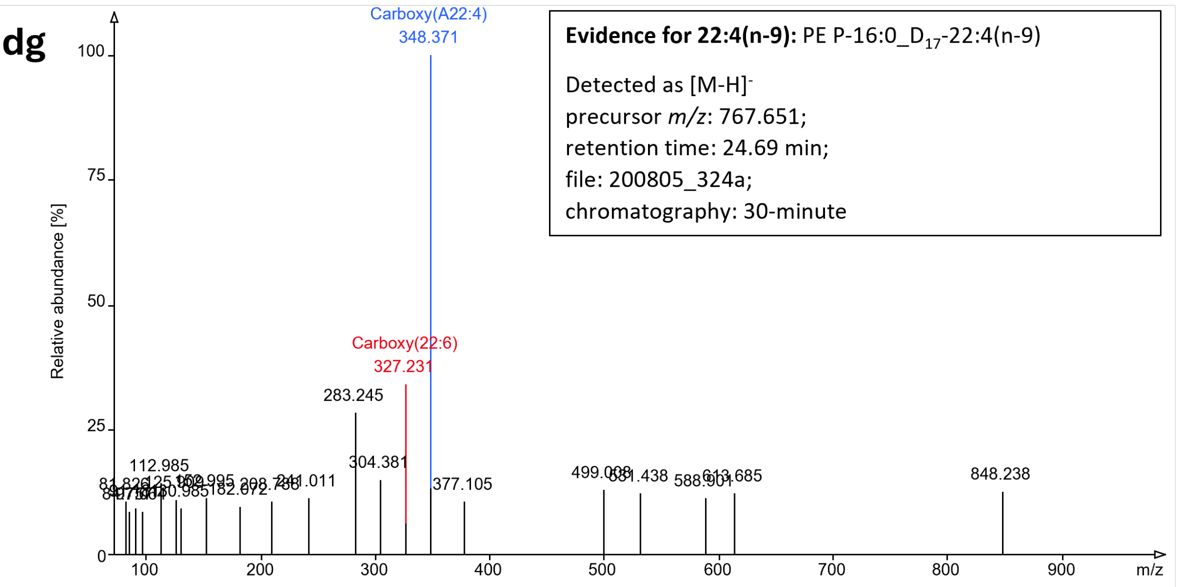

Evidence for 22:4(n-9): PE P-16:0_D_{17}-22:4(n-9)

Detected as $[M-H]^-$
precursor *m/z*: 767.651;
retention time: 24.69 min;
file: 200805_324a;
chromatography: 30-minute

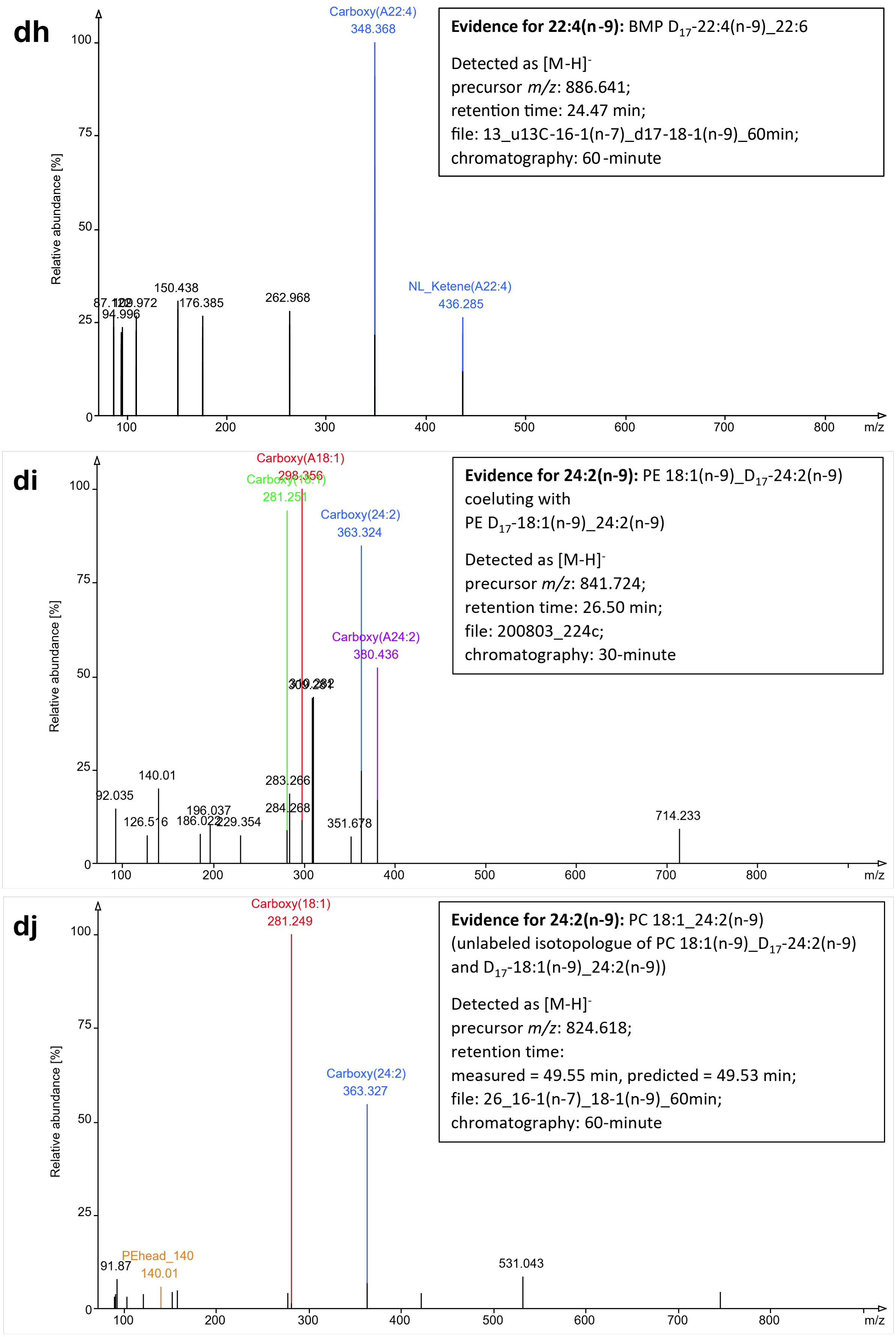
dh
Relative abundance [%]
Carboxy(A22:4)
348.368
NL_Ketene(A22:4)
436.285
150.438
176.385
262.968
94.996
m/z
Evidence for 22:4(n-9): BMP D17-22:4(n-9)_22:6
Detected as [M-H]-
precursor m/z: 886.641;
retention time: 24.47 min;
file: 13_u13C-16-1(n-7)_d17-18-1(n-9)_60min;
chromatography: 60-minute
di
Relative abundance [%]
Carboxy(A18:1)
281.251
Carboxy(24:2)
363.324
Carboxy(A24:2)
380.436
140.01
92.035
126.516
196.037
229.354
283.266
284.268
351.678
714.233
m/z
Evidence for 24:2(n-9): PE 18:1(n-9)_D17-24:2(n-9)
coeluting with
PE D17-18:1(n-9)_24:2(n-9)
Detected as [M-H]-
precursor m/z: 841.724;
retention time: 26.50 min;
file: 200803_224c;
chromatography: 30-minute
dj
Relative abundance [%]
Carboxy(18:1)
281.249
Carboxy(24:2)
363.327
PEhead_140
140.01
91.87
531.043
m/z
Evidence for 24:2(n-9): PC 18:1_24:2(n-9)
(unlabeled isotopologue of PC 18:1(n-9)_D17-24:2(n-9)
and D17-18:1(n-9)_24:2(n-9))
Detected as [M-H]-
precursor m/z: 824.618;
retention time:
measured = 49.55 min, predicted = 49.53 min;
file: 26_16-1(n-7)_18-1(n-9)_60min;
chromatography: 60-minute

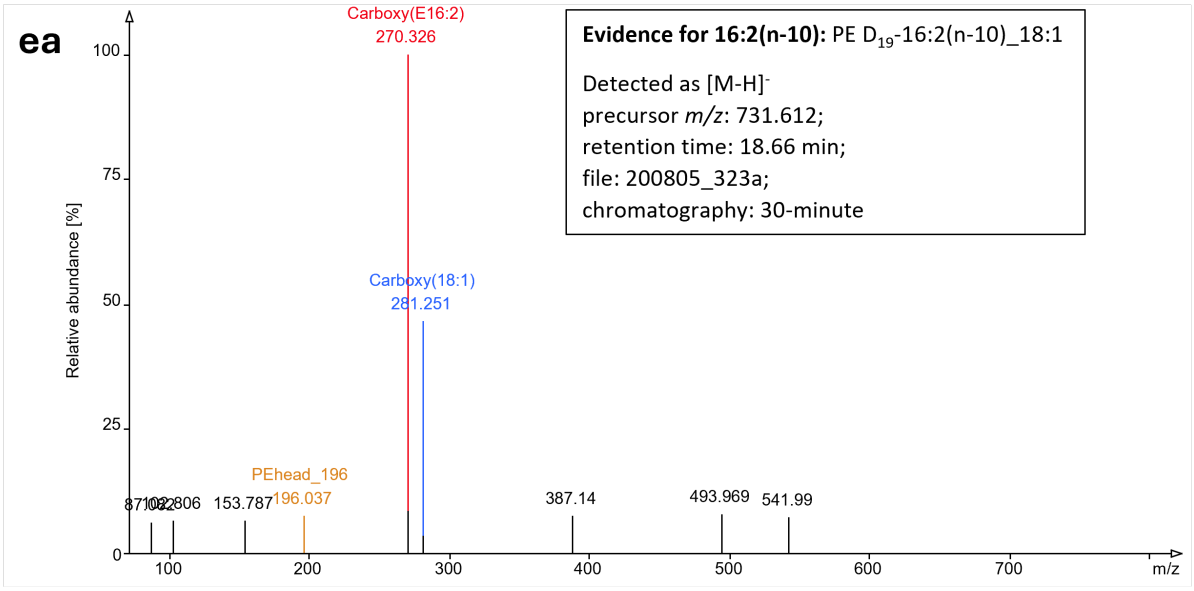
ea
Relative abundance [%]
100
75
50
25
0
Carboxy(E16:2)
270.326
Carboxy(18:1)
281.251
PEhead_196
196.037
87.02
102.806
153.787
387.14
493.969
541.99
100
200
300
400
500
600
700
m/z
Evidence for 16:2(n-10): PE D19-16:2(n-10)_18:1
Detected as [M-H]-
precursor m/z: 731.612;
retention time: 18.66 min;
file: 200805_323a;
chromatography: 30-minute

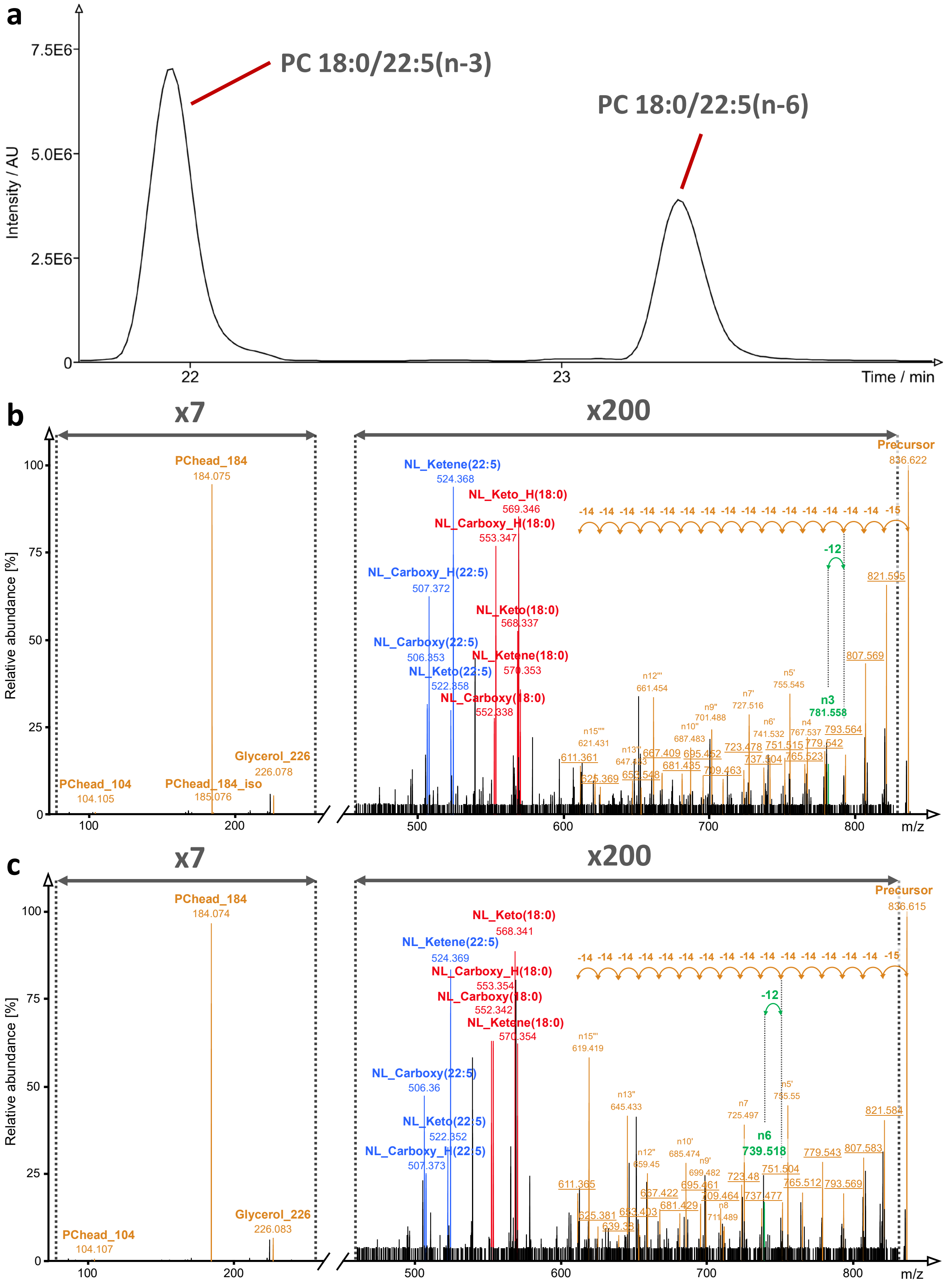

Supplementary Fig. 6: Chromatogram and EAD evidence for PC 18:0/22:5 ω-positional isomers in human plasma.
a Chromatographic separation of ω-positional isomers using the 30-minute chromatography at the example of PC 18:0/22:5(n-3) and PC 18:0/22:5(n-6) in human plasma (file: '250213_NIST_30min_IDA_EAD_pos_01'). **b** EAD spectrum acquired at 21.91 min, precursor *m/z* = 836.612 (same file origin as in subfigure **a**) shows the fragments indicative for PC 18:0/22:5(n-3). **c** EAD spectrum acquired at 23.32 min, precursor *m/z* = 836.613 (same file origin as in subfigure **a**) shows the fragments indicative for PC 18:0/22:5(n-6).

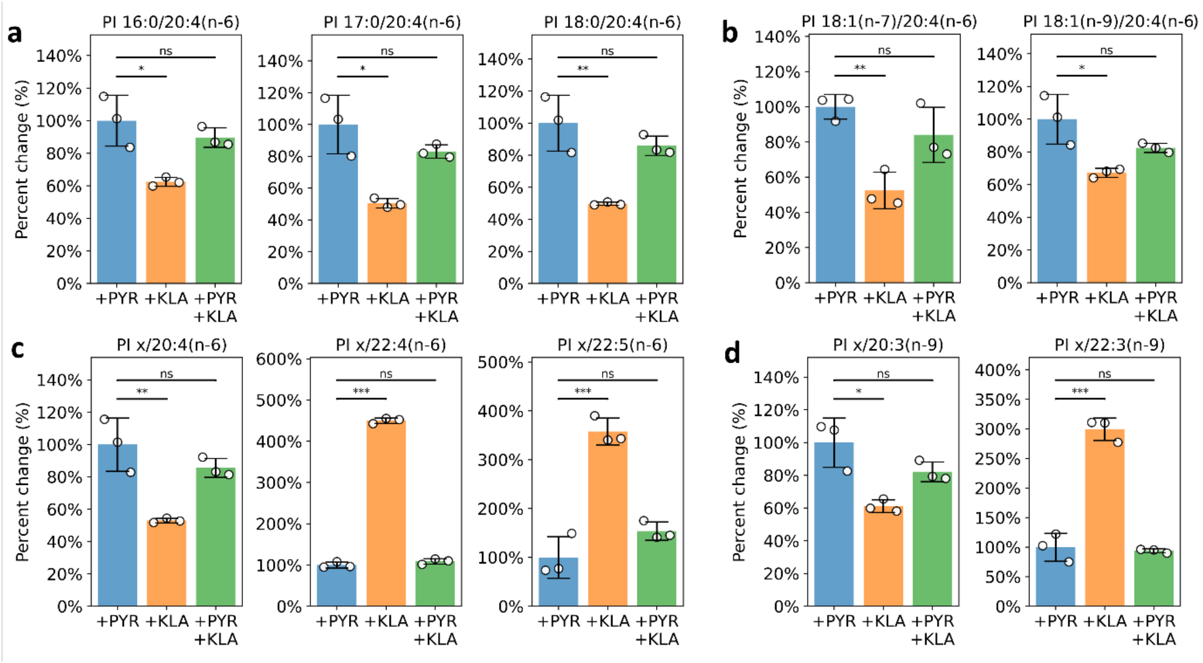

Supplementary Fig. 7: *sn*-resolved $cPLA_2$ specificity and downstream effects of $cPLA_2$ activation.
Assay conditions are as follows: (blue) $cPLA_2$ inhibitor pyrrophenone (PYR) treatment; (orange) $cPLA_2$ activation via stimulation with KLA - the chemically defined version of the toll-like receptor-4 (TLR-4) activator lipopolysaccharide; (green) PYR pretreatment followed by 24 h KLA stimulation. Measurements were performed in biological triplicates (n=3). Error bars represent the standard deviation. Irrespective of the **a**, chain length and **b**, the C=C position in the FA at the *sn*-1 position, $cPLA_2$ shows significant activity for AA. Downstream elongation and desaturation products are observed upon release of **c**, AA and **d**, MA by $cPLA_2$ activation. Source data are provided as a Source Data file.

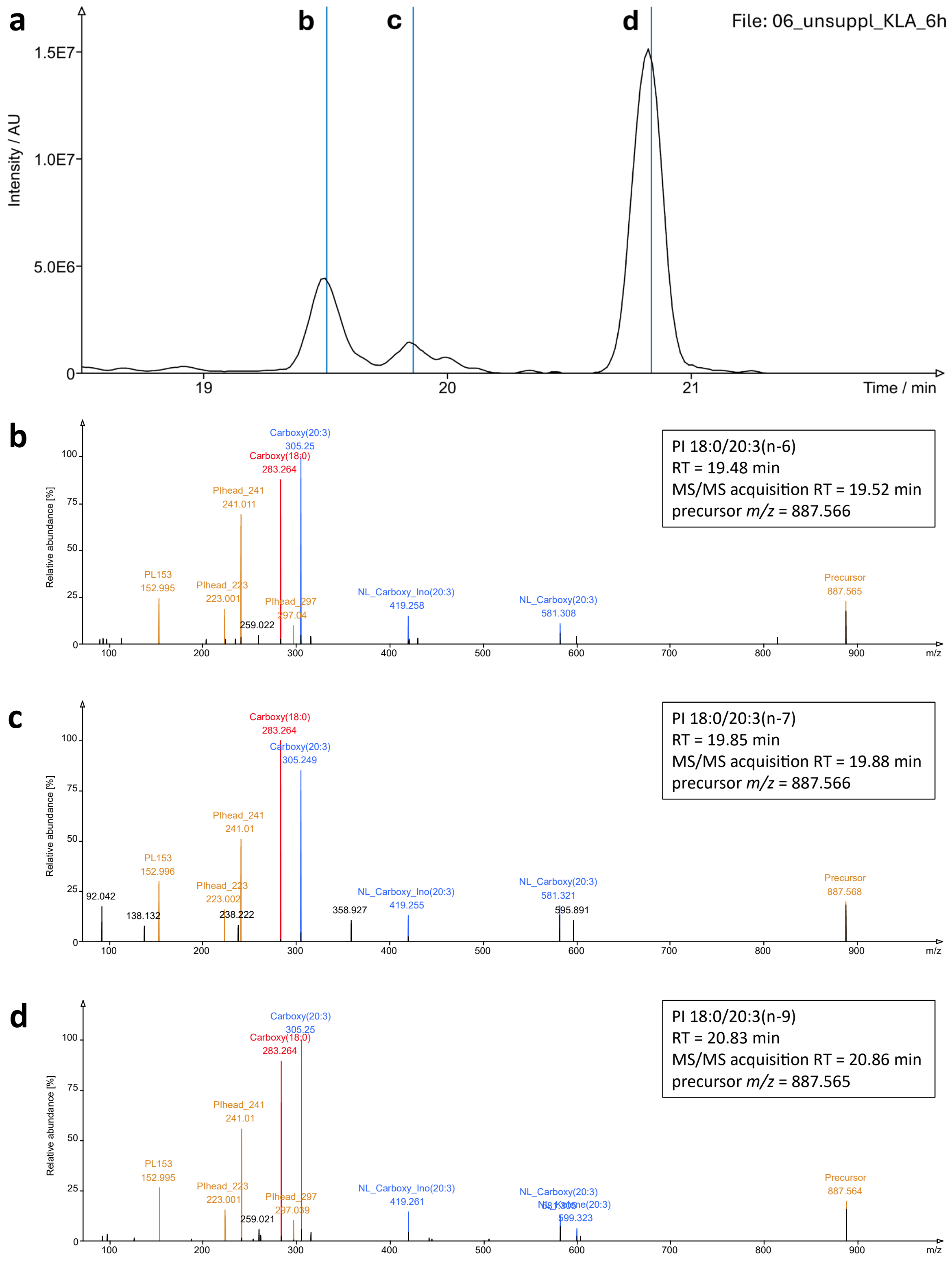

Supplementary Fig. 8: Spectral evidence of PI 18:0/20:3 at RTs specific for ω-position isomers.
a Experimental chromatogram of C=C-positional isomers of PI 18:0/20:3, observed with a 30-minute gradient after $cPLA_2$ activation via stimulation with KLA. Blue vertical lines indicate RTs at which the spectra **b-d** were acquired. The neutral loss (NL) fragments 'NL_Carboxy_Ino', 'NL_Carboxy' and 'NL_Ketene' are fragments characteristic for FAs at the *sn*-2 position in PI. Since 20:3 is consistently observed at *sn*-2, a retention time shift due to positional isomerism can be excluded.

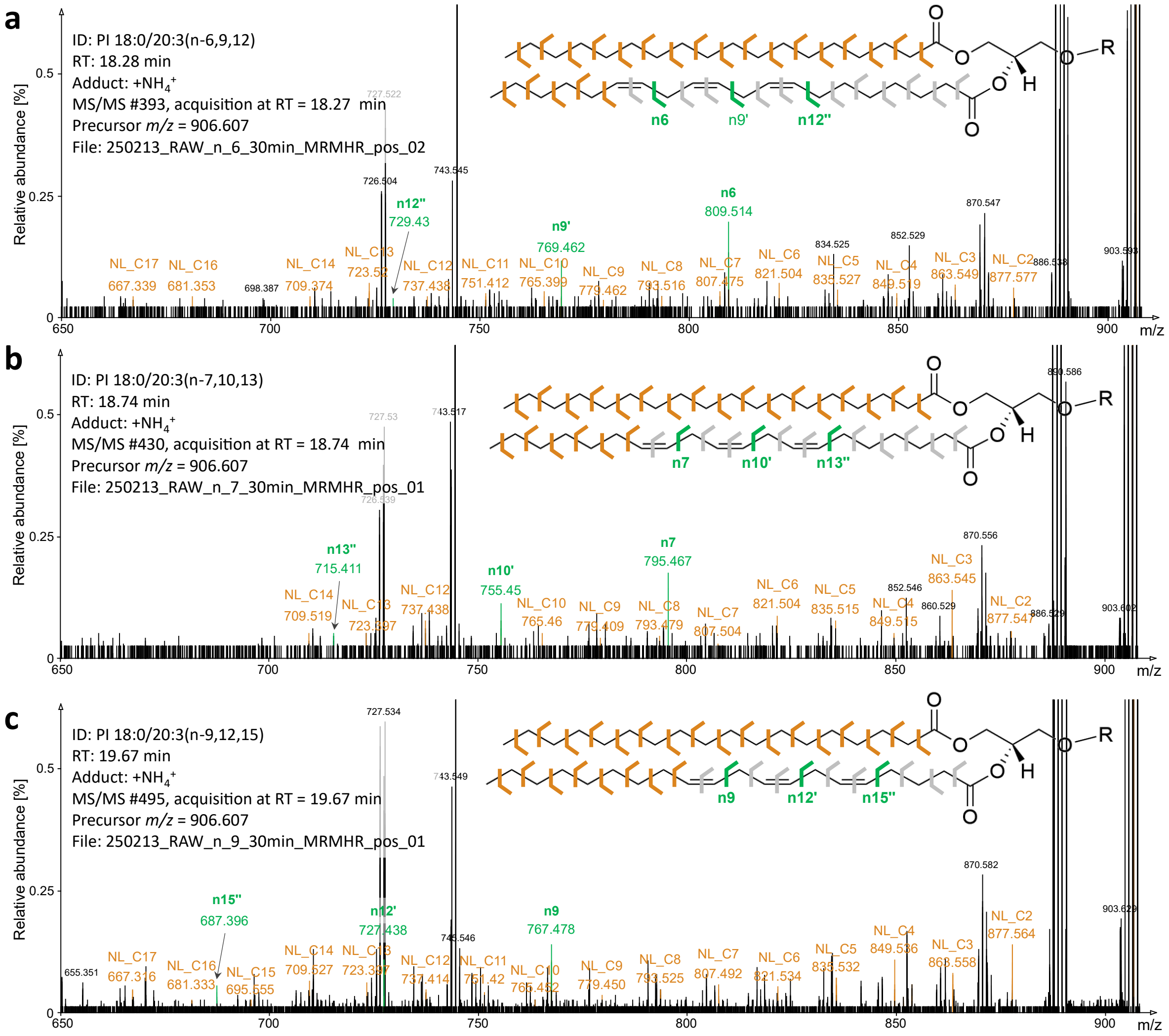

d

	PI 18:0/20:3(n-6,9,12)	PI 18:0/20:3(n-7,10,13)	PI 18:0/20:3(n-9,12,15)
RT	18.28 min	18.71 min	19.66 min
ω-C=C (identified/total)	9/9	8/9	9/9
middle C=C (identified/total)	9/9	7/9	9/9
first C=C (identified/total)	9/9	5/9	9/9

Supplementary Fig. 9: Spectral EAD evidence of C=C positions in the identified PI 18:0/20:3 isomers with EAD. RAW264.7 cells were supplemented with the native FAs 18:2(n-6), 16:1(n-7), and 18:1(n-9) to increase the abundance of the PI 18:0/20:3 isomers with the respective ω-position. Cell extracts were measured in technical triplicates (n=3) by RPLC-MS/MS in positive ion mode using EAD at 12 eV and an electron beam current of 5500 nA in MRMHR mode with an accumulation time of 215 ms. The data was analyzed with LC=CL and the annotation of C=C-specific fragments was manually confirmed. The fragment annotations starting with 'NL_C' denote neutral losses (NL) of carbohydrate chains, where the number indicates the number of carbons lost (i.e., 'NL_C2' denotes a NL of C2H5, 'NL_C3' a NL of C3H7 etc.). The fragments indicative for a C=C are NLs with two hydrogens fewer than the expected NL for a saturated chain, e.g. the 'n6' NL, which indicates an omega 6 species, involves two hydrogens fewer than 'NL_C7'. Subsequent C=C positions involve a loss of four, six, etc. hydrogens fewer and are denoted with an additional apostrophe ('), respectively (i.e., n9', n12''). **a** Evidence for the C=C-positions of PI 18:0/20:3(n-6,9,12), identified at RT=18.28 min (see also **Supplementary Fig. 3b**); **b** evidence for the C=C-positions of PI 18:0/20:3(n-7,10,13), identified at RT=18.74 min; **c** evidence for the C=C-positions of PI 18:0/20:3(n-9,12,15), identified at RT=19.67 min; **d** for each identified C=C position, the number of repeats the fragment characteristic for the respective C=C position has been identified in is listed in comparison to the total number of measurements. Notably, for each supplementation, all of the respective C=C positions could be unambiguously identified in all three triplicates.

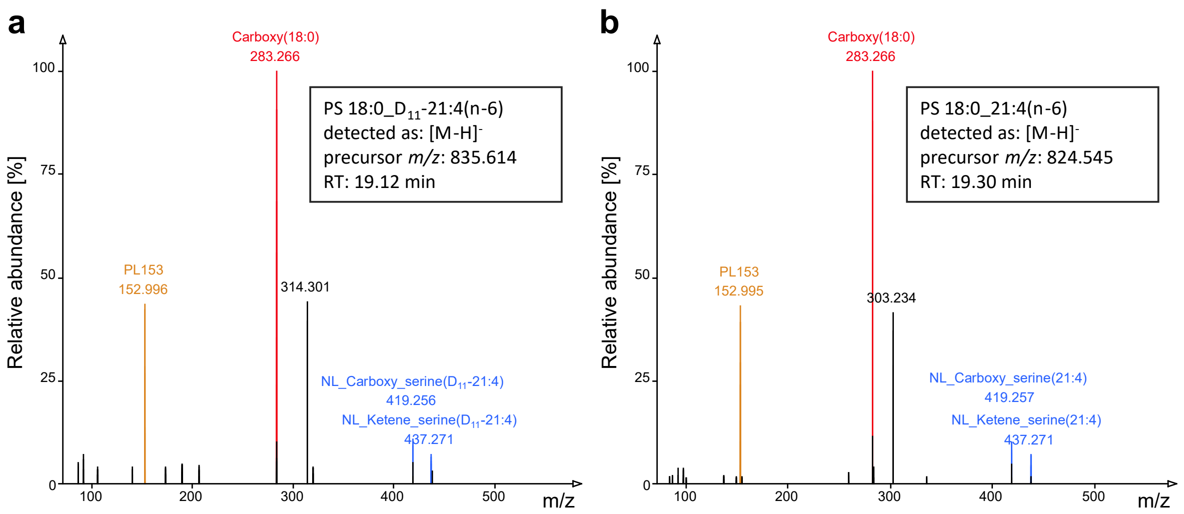

Supplementary Fig. 10: Spectral evidence for the odd-chain FA 21:4(n-6)

MS^n spectra of **a** PS 18:0_D_{11}-21:4(n-6) as well as **b** the respective unlabeled isotopologue are shown, indicating the presence of FA 21:4(n-6). Both were detected in the same MS measurement of A30 (file: 200803_226b); measured RT of PS 18:0_D_{11}-21:4(n-6) = 19.12 min; predicted RT for PS 18:0_21:4(n-6) = 19.32 min, measured RT = 19.30 min.

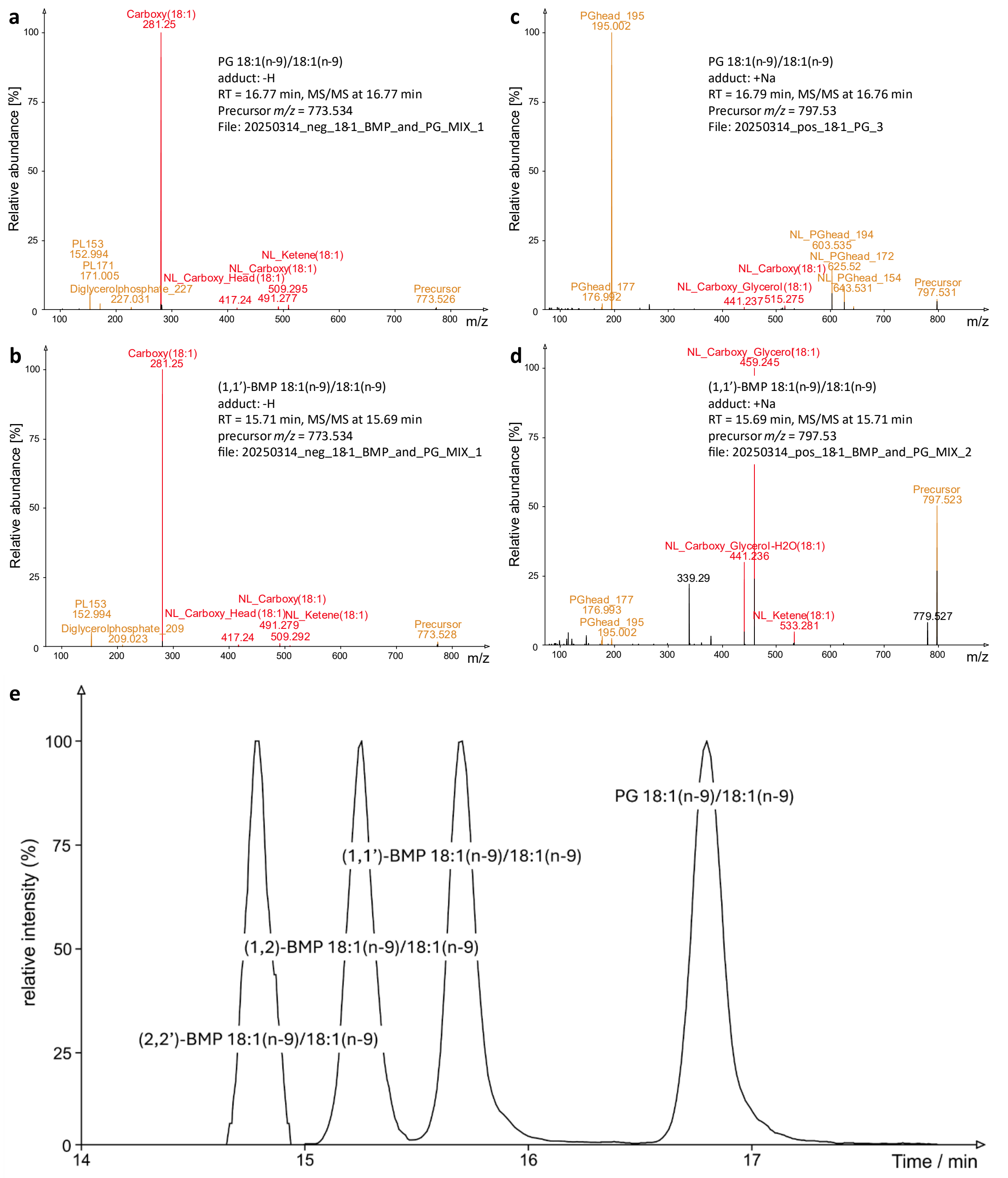

Supplementary Fig. 11: Distinction of the isomeric lipid classes BMP and PG based on measurements of authentic standards.

RPLC-MS/MS measurements of BMP and PG standards in negative and positive ion mode observed with a 30-minute gradient. MS/MS spectra in negative ion mode of **a** PG 18:1(n-9)/18:1(n-9) and **b** (1,1')-BMP 18:1(n-9)/18:1(n-9) reveal mostly identical fragmentation. In positive ion mode, the two lipid classes produce easily distinguishable spectra **c** PG 18:1(n-9)/18:1(n-9) and **d** (1,1')-BMP 18:1(n-9)/18:1(n-9). **e** The three BMP isomers (2,2')-BMP, (1,2)-BMP, and (1,1')-BMP elute significantly earlier than their PG counterpart.

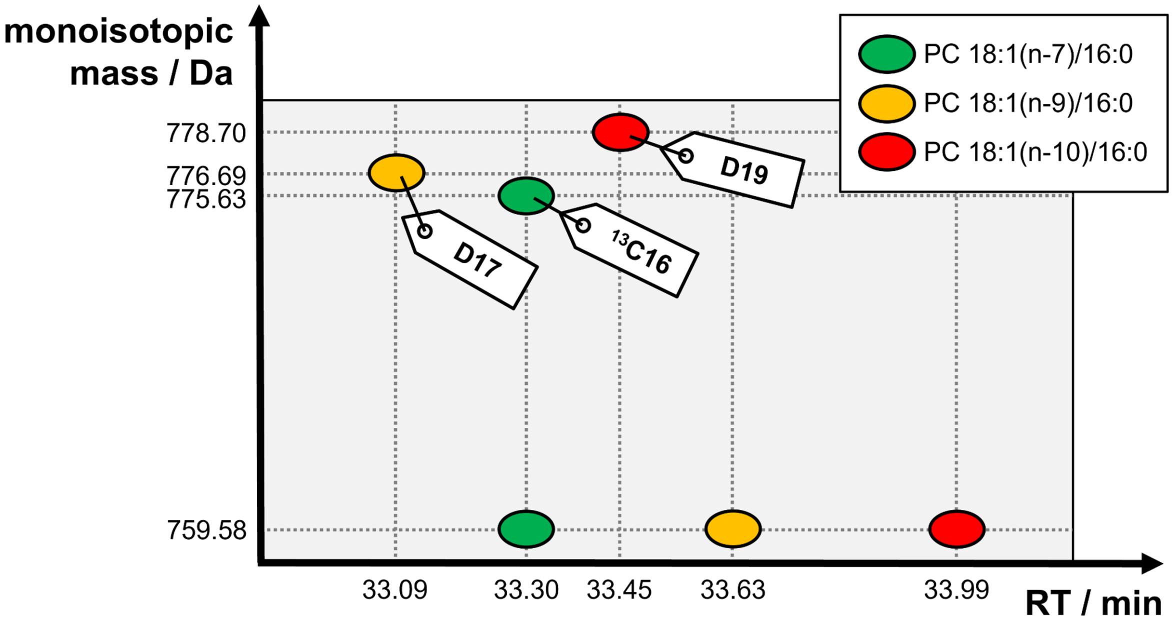

Supplementary Fig. 12: Isotope effect on chromatographic retention illustrated by authentic standards.
Measurements of authentic standards (PC 18:1(n-7)/16:0, PC 18:1(n-9)/16:0 and PC 18:1(n-10)/16:0) as well as their SIL isotopologues in a 60-minute chromatography (the chromatographic and *m/z* ranges were zoomed to show the relevant region). The deuterium labeled lipids (yellow and red) exhibit a significant effect on chromatographic retention, whereas the impact of ^{13}C labeling is negligible (green).

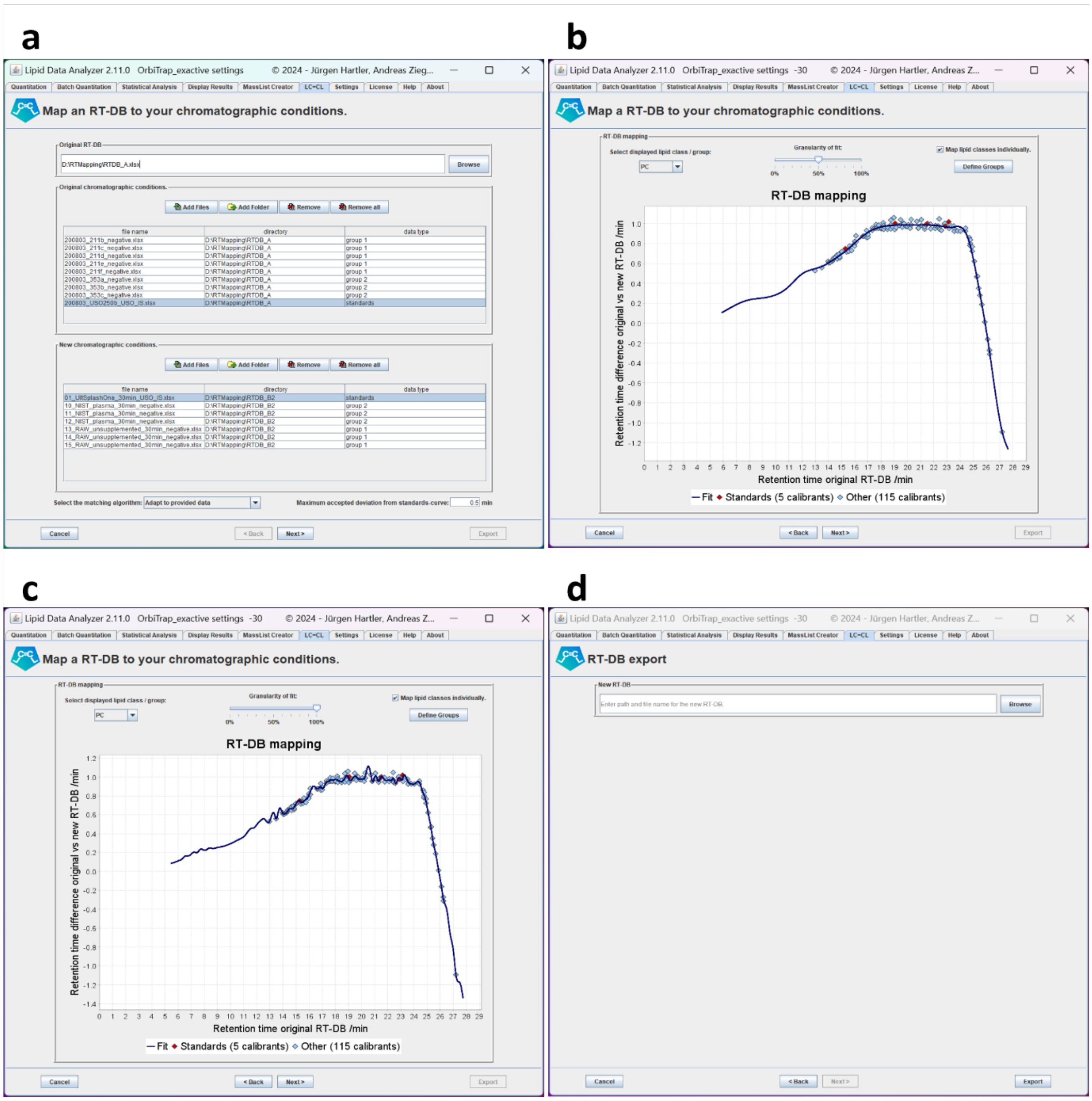

Supplementary Fig. 13: LC=CL predictor graphical user interface (GUI).

Overview of the RT-DB predictor GUI at the example of the RT mapping from RT-DB A30 to B30b. **a** GUI for the selection of the original RT-DB as well as the result files from which the anchor species for RT mapping should be retrieved. For the data type, 'standards' should be used for standards only mixtures, while any other designation can be used for other reference sample types. Matches with a higher deviation from the standards-curve than the one set for 'Maximum allowed deviation from standards-curve' will be discarded automatedly to reduce the number of false matches requiring manual removal as detailed in **Supplementary Fig. 14**. **b** The GUI displays a preview of the suggested RT mapping. With the checkbox 'Map lipid classes individually', users may select lipid (sub)class specific RT mapping, otherwise all data will be mapped by the same prediction. Any lipid classes without enough data points available (at least 3), will be mapped with a prediction originating from all data points, irrespective of the selection. The button 'Define Groups' opens a menu for defining groups for combined mappings (see **Supplementary Fig. 17**). With the slider 'Granularity of fit', users can adjust how closely the fit follows individual data points, from 100% (bin lengths of 1/150 of the original chromatography are used) to 0% (bin length corresponds to 1/15). RT ranges for individual lipid classes or groups without enough data points for the selected granularity are filled with data points from the fit of all available data combined where available. In this example, as shown in **b**, the default granularity value of 50% is a good choice. **c** For the same example, a granularity of 100% entails overfitting, but such a setting may be suitable for other chromatographic conditions. **d** Finally, the user can export the mapped RT-DB and use it for the LC=CL assignments.

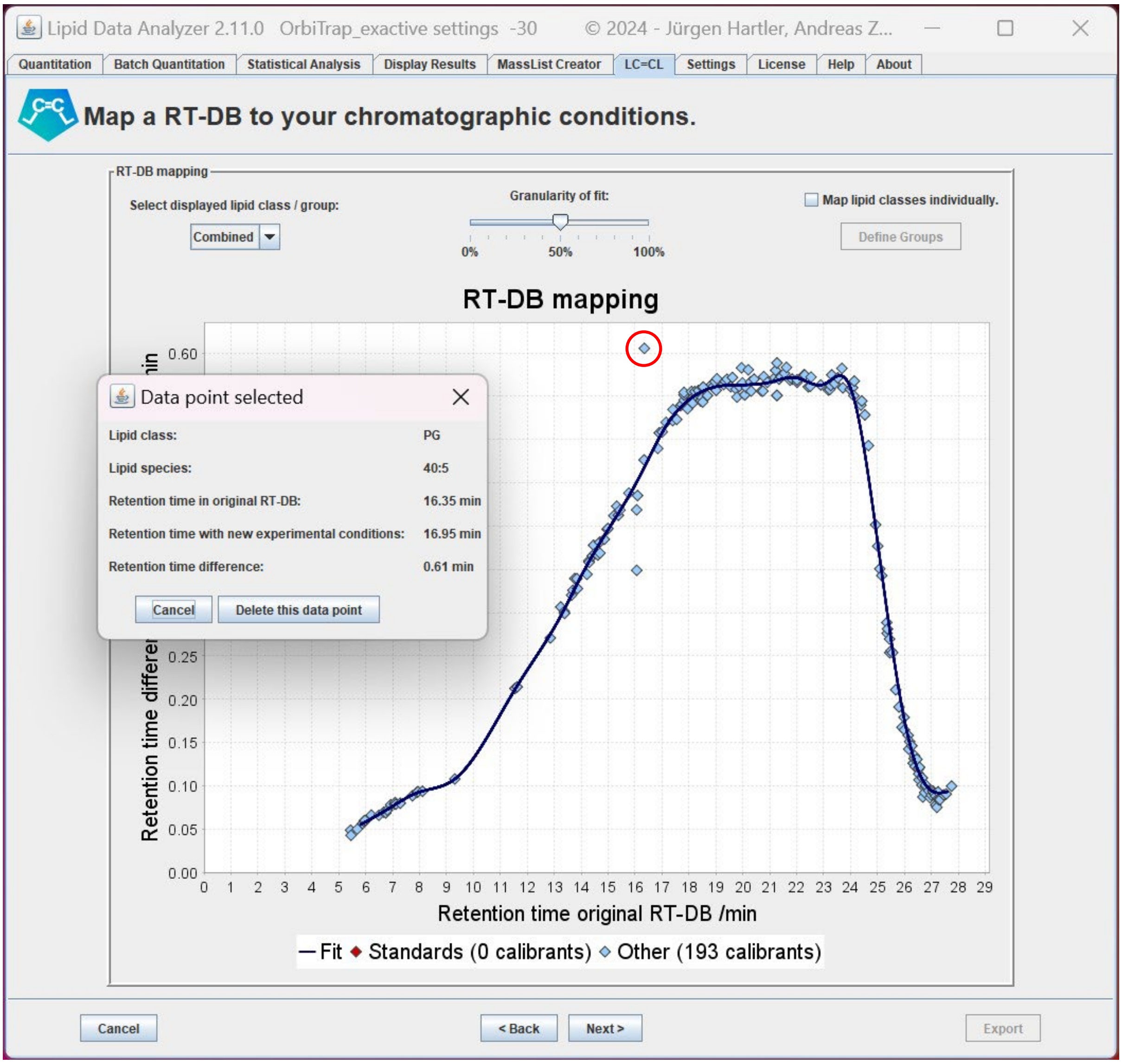

Supplementary Fig. 14: GUI for visual removal of false positives for RT mapping.
In the RT prediction example from RT-DB A30 to RT-DB B30a, two outliers can be easily detected by eyeballing. Clicking the left mouse button on such a data point prompts a dialog box for removing it as an anchor point for the RT mapping. The depicted dialog box corresponds to the false positive highlighted by a red circle.

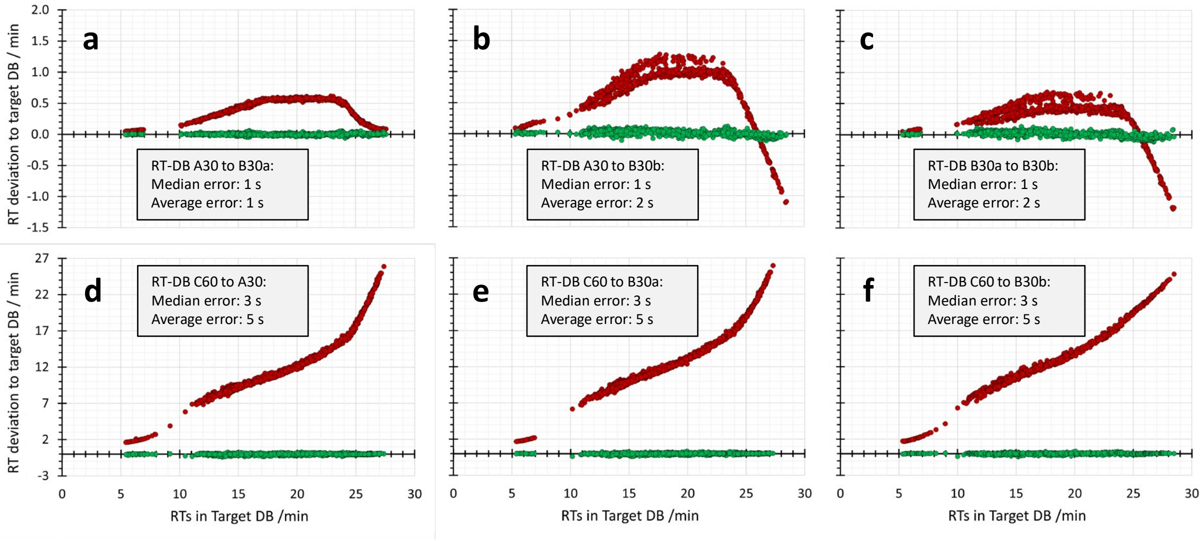

Supplementary Fig. 15: RT mapping between different RPLC-MS/MS measurements with LC=CL.
RT mapping between chromatographic conditions used in the creation of different pairs of RT-DBs. Each data point represents a lipid molecular species with an assigned ω-position that was detected in both measurements. The data series in red represents the RT deviation before applying the LC=CL RT predictor. The data series in green represents the RT error of the same lipid species after RT mapping. **a**, RT mapping from RT-DB A30 to B30a. Only the batch of mobile phase differs between these measurements of two different biological replicates. Median and average errors of one second were obtained, respectively. **b**,**c**, RT mapping from RT-DB A30 and B30a to B30b. In addition to different mobile phases, RT-DB B30b differs in the stationary phase used in the experiment. Median and average errors of one and two seconds, respectively, were achieved in both cases. **d**-**f**, RT mapping from RT-DB C60 to A30, B30a and B30b. RT-DB C60 has been created based on a 60-min gradient, while all the others featured a 30-min gradient. Median and average errors of three and five seconds, respectively, were achieved in all three cases.

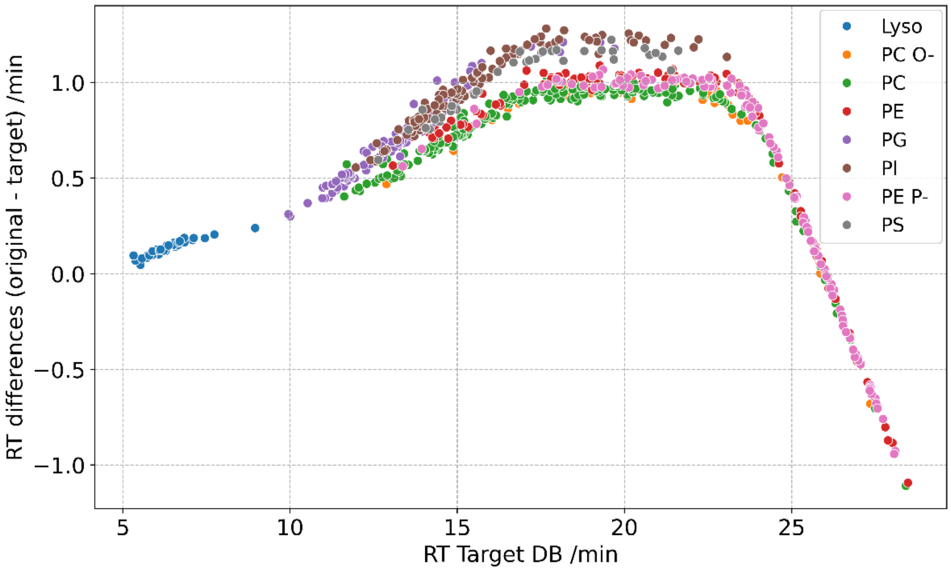

Supplementary Fig. 16: Differing effects of stationary phase batches depending on the lipid (sub)class.
Differing batch effects of the stationary phase exemplified by the RT differences between the measurements A30 and B30b (see also **Supplementary Fig. 15b**). The general term 'Lyso' summarizes all lysophospholipid classes that were analyzed (LPC, LPE, LPG, LPI and LPS), which exhibited similar RT differences between the two measurements.

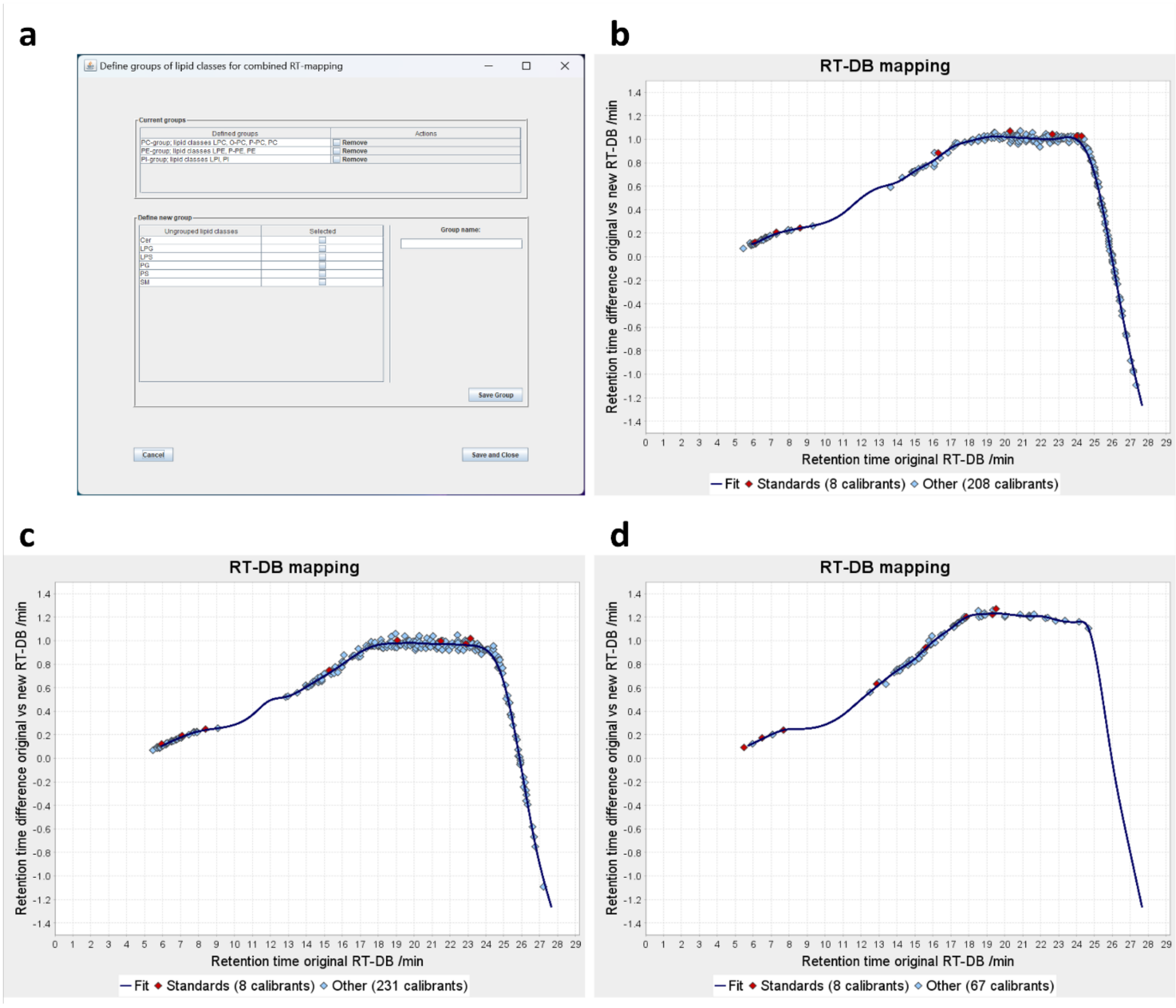

Supplementary Fig. 17: GUI for the definition of groups subject to similar batch-effects on chromatographic retention. LC=CL provides the option of defining groups of lipid (sub)classes for instances where common trends in batch-effects on retention behavior among different lipid classes are observed in the RT predictor GUI. This is shown at the example of the RT mapping of RT-DB A30 to B30b. This is a convenient way to increase the number of anchor points, in particular for (sub)classes typically observed at low abundance, and consequently improve the accuracy of RT mapping. **a** GUI for the definition of lipid (sub)class groups: At the bottom panel, lipid (sub)classes can be added to groups, which are displayed at the top panel. By this selection, individual RT-mappings of each group will be created; **b** preview of RT-mapping based on a group consisting of LPE, PE and PE P-; **c** preview of RT-mapping based on a group consisting of LPC, PC, PC O- and PC P-; **d** preview of RT-mapping based on a group consisting of LPI, and PI.

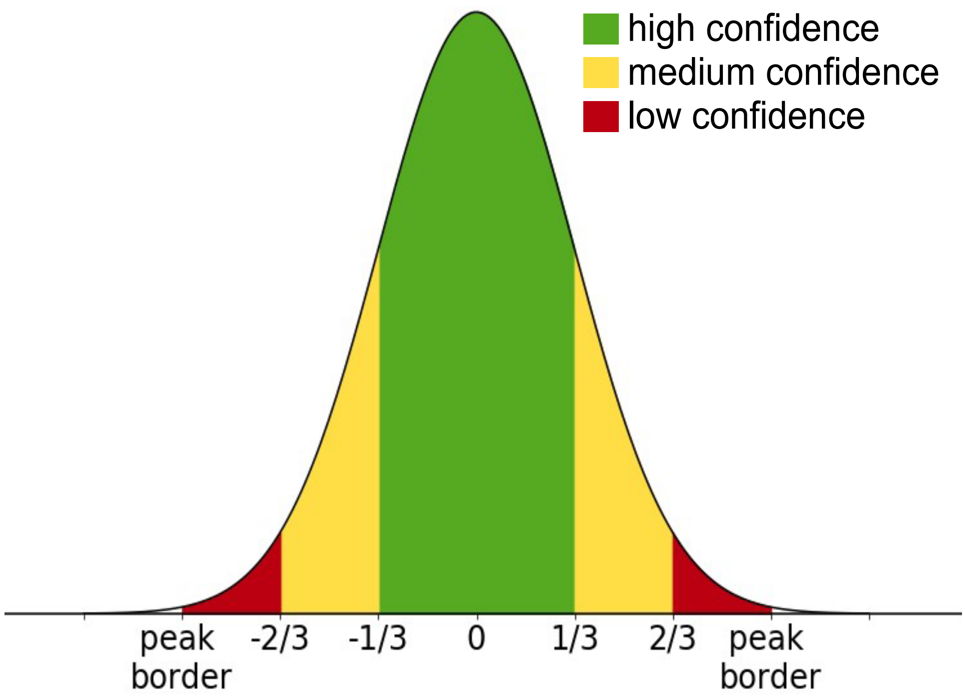

Supplementary Fig. 18: Scheme for automated annotation of ω-positions.

The algorithm considers all MS^2 confirmed molecular species for ω-position assignment, where predicted RTs of the respective identified species fall within the borders of the MS^1 chromatographic peak. The 'high confidence' interval, shaded in green, is the range from the peak's apex to one-third of the distance to the peak border (using the shorter distance for asymmetric peaks). The 'medium confidence' interval, shaded in yellow, extends to two-thirds of the distance, and the 'low confidence' interval, shaded in red, covers the RT range beyond two-thirds. If a predicted RT falls within the high confidence interval, the corresponding RT-DB entry is automatically assigned, provided there are no other ω-predictions that match equally well. If the predicted RT is in the medium or low confidence intervals, it can still be assigned automatically if it is within a user-defined threshold (we used 12 seconds) and there are no other possible ω-position assignments. If no automated peak assignment is possible, the remainder of predictions within the peak borders are offered as suggestions for potential manual evaluation in the GUI.

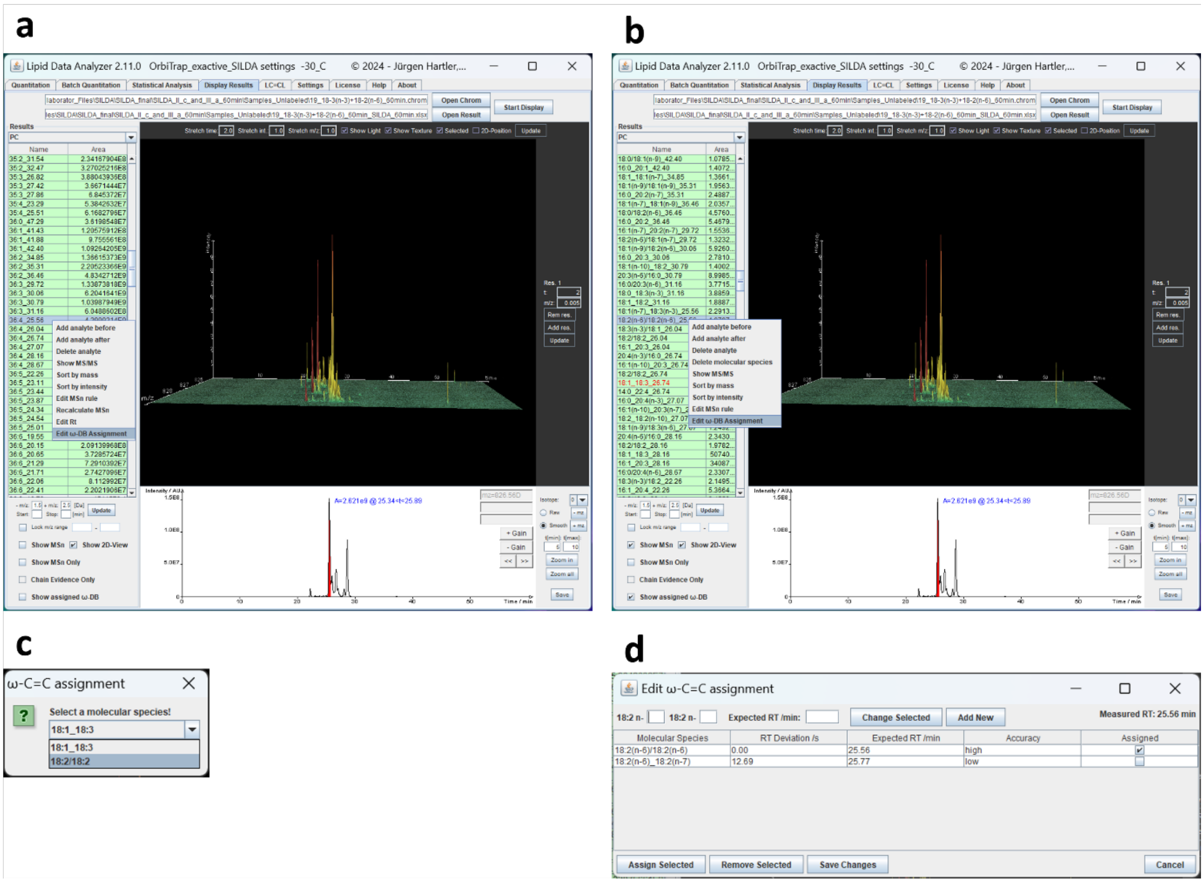

Supplementary Fig. 19: GUI for selecting or discarding suggested ω-position information.
In LDA's spectral viewer, the detected species can be either displayed at **a** the lipid species level or at **b** the lipid molecular species level. Both views allow for editing of ω-positions with a right-click on a chosen lipid. A lipid species may encompass several different lipid molecular species. Therefore, at the species level view, the user may be prompted with a popup (shown in **c**) to choose the corresponding lipid molecular species. Then, the 'Edit ω-C=C assignment' panel (see **d**) is displayed where ω-positions for the lipid molecular species can be assigned from a list of all suggested RT matches based on actual peak borders from the RT-DB used in the LC=CL quantification. Alternatively, the GUI also allows for manual correction of ω-position assignments.

Supplementary Note 1: RT mapping between different RPLC-MS/MS measurements with LC=CL.

The developed machine learning algorithm calibrates the selected RT-DB to the new elution profile of the current chromatographic conditions. The anchor species required by the RT prediction algorithm may be provided by a set of (internal) standards, and/or lipid species identified in any biological sample. In this study, we employed measurements of the UltimateSPLASH™ ONE internal standard mixture (USO) available from Avanti Polar Lipids and RAW264.7 cell extracts, using a different biological replicate in each experiment to mimic a real-life application. For confident FA-specific ω-position identifications, calibration errors of less than ten seconds are required, as this is the typical RT difference to separate the ω-position isomers at our 30-minute gradient (see **Supplementary Fig. 4**). Our machine learning algorithm achieved median and average errors as low as 1-2 seconds for everyday use cases, and even for the calibration from a 60-minute to a 30-minute gradient, the average errors were below 5 seconds (**Supplementary Fig. 15**). Accordingly, reliable ω-position identification is effortlessly possible by our approach.

To obtain these errors (measures for accuracy and robustness), we utilized the four experimentally obtained RT-DBs. Based on one RT-DB, the machine learning algorithm predicted the RTs of the other chromatographic conditions, and these values were compared to the experimentally derived RTs (**Supplementary Fig. 15** and **Supplementary Data 5**). For the everyday use-case of differing batches of mobile phase (mapping of RT-DB A30 to B30a, **Supplementary Figure 15a**), our algorithm achieved mean and average errors of one second. We found that an internal standard mixture alone is sufficient for calibration, which should be present anyway for quantification purposes (**Supplementary Data 6**).

Next, we tested the effect of differing stationary phase. We found that batch effects of the RPLC column have differing effects on lipid species depending on their head group, i.e. their class (**Supplementary Fig. 16**). For such cases, the fully automated RT mapping procedure as described in the previous paragraph should be tailored to individual lipid classes, or user-defined groups of classes with similar effects on retention behavior (supported by LC=CL, see **Supplementary Fig. 17**). As a rule-of-thumb, each group should be covered by at least seven evenly spread anchor points. Thus, if such anchor points cannot be provided by a mixture of standards, we recommend additionally supplying LC=CL with the measurements of a complex biological sample. LC=CL generates a new mapped RT-DB, which can be used for all follow-up experiments. Notably, despite different stationary phases and class-specific RT trends, our RT-DB mapping algorithm achieved remarkably low mean and average errors of one and two seconds, respectively (see mappings of the RT-DBs A30 and B30a to the RT-DB B30b **Supplementary Fig. 15b,c**).

Finally, we tested our algorithm's performance based on mapping a 60-minute gradient to previously used 30-minute gradients (RT-DB C60 to RT-DBs A30, B30a and B30b); encompassing also different mobile and stationary phases. Here, we obtained mean and average errors as low as three and five seconds, respectively (**Supplementary Fig. 15d-f**). This accuracy is satisfactory, as we were able to separate the peak maxima of identical lipid molecular species differing by a single ω-position (e.g. n-9 and n-10) on average by ten (30-minute gradient) and eighteen seconds (60-minute gradient), respectively.

These results conclusively demonstrate that our developed machine learning algorithm can accurately calibrate our experimentally verified RT-DBs toward any experimental setups, making as such ω-position information easily accessible.

To provide a wide coverage, we aggregated the four experimentally obtained RT-DBs into a comprehensive curated RT-DB containing 2408 phospholipid species. We provide this RT-DB with the elution profile for the 30-minute gradient (RT-DB_30min) and the 60-minute gradient (RT-DB_60min)

used in this study, to ensure highly reliable RT mapping using the LC=CL RT predictor for different use-cases.
