# Supplementary material for: Computationally unmasking each fatty acyl C=C position in complex lipids by routine LC-MS/MS lipidomics
Source: Nat Commun. 2025 Aug 11;16:7277. doi: 10.1038/s41467-025-61911-x (PMC12340080; doi:10.1038/s41467-025-61911-x)
Supplement: Supplementary file 2 — Description of Additional Supplementary Files [file 41467_2025_61911_MOESM2_ESM.docx]

**Description of Additional Supplementary Files**

File Name: Supplementary_Data_1.xlsx

Description: Overview of the lipid molecular species and FAs in LC=CL’s aggregate RT‑DB.

File Name: Supplementary_Data_2.xlsx

Description: List of novel species. 'Novel' corresponds to not being present in either the HMDB, RefMet or LMSD database.

File Name: Supplementary_Data_3.xlsx

Description: Comparison of lipid identifications at the ω-position level between LC=CL and the three orthogonal methods PB, EAD, and OzID.

File Name: Supplementary_Data_4.xlsx

Description: Result of manual curation at the example of RT-DB B30a.

File Name: Supplementary_Data_5.xlsx

Description: RT mapping values as depicted in Supplementary Fig. 15.

File Name: Supplementary_Data_6.xlsx

Description: RT mapping of RT-DB A30 to RT-DB B30a using only the USO standard mix or only the cell extract.
